# Non-Pharmacological Interventions to Improve Chronic Disease Risk Factors and Sleep in Shift Workers: A Systematic Review and Meta-Analysis

**DOI:** 10.3390/clockssleep3010009

**Published:** 2021-01-28

**Authors:** Meagan E Crowther, Sally A Ferguson, Grace E Vincent, Amy C Reynolds

**Affiliations:** 1 The Appleton Institute, CQUniversity, 44 Greenhill Road, Wayville, SA 5034, Australia; sally.ferguson@cqu.edu.au (S.AF.); g.vincent@cqu.edu.au (G.EV.); 2 School of Health, Medical and Applied Sciences, CQUniversity Australia, Adelaide Campus, Wayville, SA 5034, Australia; 3 Flinders Health and Medical Research Institute (Sleep Health)/Adelaide Institute for Sleep Health (AISH): A Flinders Centre of Research Excellence, College of Medicine and Public Health, Flinders University, Bedford Park, SA 5042, Australia; amy.reynolds@flinders.edu.au

**Keywords:** occupational health, chronic disease, sleep, light therapies, health promotion, work schedule tolerance, complementary therapies, health behaviour, shift work

## Abstract

Shift work is associated with adverse chronic health outcomes. Addressing chronic disease risk factors including biomedical risk factors, behavioural risk factors, as well as sleep and perceived health status, affords an opportunity to improve health outcomes in shift workers. The present study aimed to conduct a systematic review, qualitative synthesis, and meta-analysis of non-pharmacological interventions targeting chronic disease risk factors, including sleep, in shift workers. A total of 8465 records were retrieved; 65 publications were eligible for inclusion in qualitative analysis. Random-effects meta-analysis were conducted for eight eligible health outcomes, including a total of thirty-nine studies. Interventions resulted in increased objective sleep duration (Hedges’ g = 0.73; CI: 0.36, 1.10, *k* = 16), improved objective sleep efficiency (Hedges’ g = 0.48; CI: 0.20, 0.76, *k* = 10) and a small increase in both subjective sleep duration (Hedges’ g = 0.11; CI: −0.04, 0.27, *k* = 19) and sleep quality (Hedges’ g = 0.11; CI: −0.11, 0.33, *k* = 21). Interventions also improved perceived health status (Hedges’ g = 0.20; CI: −0.05, 0.46, *k* = 8), decreased systolic (Hedges’ g = 0.26; CI: −0.54, 0.02, *k* = 7) and diastolic (Hedges’ g = 0.06; CI: −0.23, 0.36, *k* = 7) blood pressure, and reduced body mass index (Hedges’ g = −0.04; CI: −0.37, 0.29, *k* = 9). The current study suggests interventions may improve chronic disease risk factors and sleep in shift workers; however, this could only be objectively assessed for a limited number of risk factor endpoints. Future interventions could explore the impact of non-pharmacological interventions on a broader range of chronic disease risk factors to better characterise targets for improved health outcomes in shift workers.

## 1. Introduction

In a modern, 24/7 world, shift work has become a ubiquitous part of the workforce. While work schedules vary across different occupations, locations and contexts, shift work can be defined as “work [that] usually encompasses work time arrangements outside of conventional daytime hours, which includes fixed early morning, evening, and night work, as well as roster work and rotating three shift work” [1]. Whilst shift work benefits the economy and workplace flexibility, a substantial body of evidence now highlights the health costs of shift work for individual workers. Shift work is associated with an increased risk of numerous chronic diseases [2,3]. Chronic diseases are defined as non-communicable, long-lasting disease with persistent effects [4]. Specifically, recent studies have demonstrated that shift workers are at increased risk of cardiovascular disease [5,6,7,8,9,10,11], prostate cancer [12,13,14,15], metabolic syndrome [7,16,17,18,19,20,21], breast cancer [7,22,23,24] and stroke [8,25,26,27].

It is likely that the increased chronic disease risk seen in shift workers is a result of a complex interplay between sleep loss, circadian misalignment, exposure to light at night and altered health behaviours [1,28,29,30]. As shown in Table 1, both biomedical risk factors and poor health behaviours (known as behavioural risk factors) are associated with the development of chronic diseases. Biomedical risk factors are bodily states, often influenced by behavioural risk factors, that increase an individual’s risk of chronic disease. Biomedical risk factors include obesity, high blood pressure, dyslipidemia, and abnormal blood glucose [31]. The Australian Institute of Health and Welfare (AIHW) defines behavioural risk factors as modifiable behaviours which influence an individual’s health including nutritional intake, physical activity, tobacco, and alcohol consumption [31]. Collectively, these biomedical and behavioural risk factors can be considered chronic disease risk factors.

In addition to the risk factors outlined in Table 1, sleep loss and poor sleep quality are also associated with an increased risk of chronic diseases including diabetes [32,33,34], obesity [32,33,34], metabolic syndrome [32,33], cardiovascular disease [32,35,36,37], chronic kidney disease [38] and osteoporosis [39]. Beyond this, perceived health status has some capacity to identify individuals at risk of poorer future health outcomes [40,41,42]. Together, chronic disease risk factors, including biomedical and behavioural risk factors, sleep and perceived health status provide insight into future chronic disease risk, and by extension, areas for potential management prior to disease onset.

Given the increased risk of chronic disease associated with shift work, an understanding of beneficial interventions aimed at improving chronic disease risk factors in these employees is critical. To date, various interventions have focused on improving health and safety outcomes in shift workers. A previous literature review by Neil-Sztramko et al. [43] qualitatively reviewed 44 studies that utilised pharmacological and non-pharmacological interventions to improve indicators of chronic health effects in rotating and night shift workers. This review found that non-pharmacological interventions such as fast forward-rotating shifts, timed use of bright light and light-blocking glasses, and targeting health behaviours including physical activity and diet yielded favourable outcomes for shift workers. Pharmacological interventions were largely not efficacious. These findings suggest that non-pharmacological interventions should be implemented to improve chronic disease risk factors for shift workers. However, the review [43] did not quantitatively examine the effect of specific interventions on chronic disease risk factors, or on sleep outcomes. Such information is crucial for identifying and managing early risk before disease negatively impacts worker quality of life.

The use of quantitative methods for examining the effect of non-pharmacological interventions on chronic disease risk factors, sleep and perceived health status is necessary to inform evidence-based, effective interventions to improve health outcomes in shift workers. The primary objective of the current systematic review and meta-analysis was to examine non-pharmacological interventions aimed at improving one or more chronic disease risk factors, sleep and/or perceived health status in shift workers. The review addresses two critical research questions:

(1) Which non-pharmacological interventions have been used to improve chronic disease risk factors and sleep in shift workers?
(2) Which non-pharmacological interventions are most effective for improving chronic disease risk factors and sleep in shift workers?

Addressing these research questions will allow us to provide empirically-based recommendations for shift workers, and identify important further research opportunities.

## 2. Methods

### 2.1. Eligibility Citeria

Studies were eligible for inclusion if they investigated a non-pharmacological intervention conducted with shift workers aimed at improving one or more chronic disease risk factors (see Table 1), sleep outcome or measure of perceived health status. Only studies reporting an intervention with shift workers were eligible for inclusion (day-only workers were excluded). Studies that included both shift workers and day-only workers were eligible for inclusion if the results from shift workers were reported separately. Studies of shift workers who worked extended hours or on-call shifts were eligible for inclusion in the systematic review but not for meta-analysis as outcomes are not directly comparable due to extended working hours likely presenting an additional risk to health [44,45].

Any non-pharmacological interventions were eligible for inclusion. Measures could be self-report or objective measures, but self-reported sleep measures were assessed separately to objective measures to account for possible differences in these measures (e.g., self-report overestimation) [46,47]. In relation to perceived health status, only validated measures of perceived health status or those adapted from validated measures were eligible. Health measures that asked participants their perception of the impact of shift work on their health were excluded.

Studies that provided interventions to shift workers with diagnosed pathologies (e.g., hypertension or shift work disorder) were excluded. Interventions that were conducted in a laboratory were excluded, as the present review aimed to investigate interventions in ecological settings. Interventions of any duration were included.

### 2.2. Information Sources and Search Strategy

The systematic review and meta-analysis intended to follow Preferred Reporting Items for Systematic Reviews and Meta-Analyses (PRISMA) guidelines [48]; however, the review protocol was not pre-registered. To identify relevant intervention studies, systematic literature searches of seven electronic databases (Embase, MEDLINE, Cochrane database, Pubmed, PsychINFO, Web of Science and CINAHL) were conducted. Database selection aligned with recommendations for optimal search strategies [49]. No date restrictions were applied to searches in order to identify all relevant studies, regardless of year of publication. Initial searches were conducted on 24 September 2019, followed by an updated search conducted on 18 November 2020. The full record of search strategies used for each search is available in Supplementary Material 1. Reference lists of included studies and Google Scholar were searched for grey literature to identify studies not captured by the original database search.

### 2.3. Study Selection

Titles and abstracts were screened against eligibility criteria and relevance to the review. Publications that were thought to meet the criteria were read in full by two authors (M.E.C. and A.C.R.) to determine eligibility. All papers were independently reviewed by both authors for eligibility and any discrepancies discussed. It was agreed that any discrepancies that could not be resolved by discussion would be adjudicated by a third researcher (S.A.F.); however, this situation did not arise.

### 2.4. Data Collection Process

Data were extracted from the articles using a pre-defined modified Cochrane data extraction sheet [50] by M.E.C. The variables extracted from each included study were: first author name, publication date, intervention design, participant characteristics (sex, age), study design, occupation, type of shift work schedule, duration of intervention, outcome measure of interest and results of studies. Where further details or clarification were needed for meta-analysis, authors were contacted wherever possible.

### 2.5. Risk of Bias in Individual Studies

Quality assessment and risk of bias assessment were conducted using the Downs and Black tool 1998 [51]. This tool was chosen as it can be used with both randomised and non-randomised studies, both included within this review. Further, the Downs and Black [51] tool has also been used for quality assessment in previous systematic reviews in shift-working populations [8] and critical reviews of interventions targeting shift workers [43]. Item 27 was modified from “Did the study have sufficient power to detect a clinically important effect where the probability value for a difference being due to chance is less than 5%?” to “Was a power analysis performed?” 0 = no or unable to determine, 1 = yes, as in previous studies [52]. This response structure aligns with the response style for the Downs and Black tool. All studies were evaluated individually by the first author and a random sample of ~20% (*n* = 12) was evaluated by the senior author. Comparisons between the scores of the two investigators were then compared and an agreement percentage calculated to ensure quality of evaluations.

### 2.6. Summary Measures

The principal summary measure used throughout the review was standardised mean difference wherever possible. Further, studies that reported statistical significance were reported qualitatively in this way.

### 2.7. Synthesis of Results

Meta-analyses were conducted if there were at least three eligible studies reporting an outcome of interest [53]. Further, studies included for meta-analysis were those that used similar methods for measuring the outcome of interest in order to allow for meaningful interpretation of meta-analytic results (e.g., alcohol consumption grams/per day and “alcohol risk scores” were not considered sufficiently similar measures).

For studies where insufficient information was reported in the published manuscript, two attempts were made to contact the corresponding authors, where contact details were available, between 20 July 2020 and 10 August 2020. Following attempts to contact authors, every effort was made to calculate missing data, in accordance with Cochrane guidelines [54]. For studies that only reported a *p*-value, mean difference was used to obtain a t-value, which was then converted to standard error, and standard deviation. Studies which presented means within figures and were of sufficient quality were processed through Plot Digitizer [55] in order to allow means and standard deviations to be extracted from figures.

Applying these approaches for calculating missing information resulted in the inclusion of more studies and thus minimised possible biased meta-analytic estimates resulting from missing data [56]. A detailed outline of statistical procedures used for each study is available upon request.

Where studies were a controlled trial, the effect size for the difference between the control and intervention group was calculated, including studies between worksites (e.g., firefighters at different stations). However, studies that utilised a control group that was not in the same occupation (e.g., participants worked in two different types of manufacturing) were assessed as within group pre-post intervention only, to avoid confounding from occupational effects. Where the study design was within groups, the effect size was calculated based on the change from baseline to follow-up period. Where there were insufficient data reported from a control group, only the intervention group data were included for meta-analysis (e.g., baseline to follow up) to allow for calculation of standard deviation.

In studies where two intervention groups were present, with one common control group, the common control group was divided by two and half the control group sample was used in the meta-analysis for each of intervention groups [54].

To allow for direct comparison of sleep duration across as many studies as possible and to allow for a clear interpretation of the effect of intervention on overall sleep duration, the decision was made to use average sleep duration effect size in meta-analysis. For calculation of total average sleep duration, effect sizes of all reported sleep durations were calculated and then combined using the Borenstein formula [57] using the *MAd* package [58] in R 3.6.2 [59]. Thus, a study that reported sleep duration after night shift, morning shift and afternoon shift was combined to provide one overall estimate of total sleep duration. In addition, studies that presented outcomes by groups which were not directly related to the intervention (e.g., age or marital status) were also combined, using the method described above, to allow for the accurate representation of the entire study and avoid attributing excessive weight to studies by including outcomes independently. Finally, in studies that measured variables on an inverse scale (e.g., higher score = worse sleep), data were transformed by subtracting the mean value for the total possible scale score to ensure the direction of scale was consistent with other studies [54]. 

Eight separate meta-analyses were performed. Meta-analyses were conducted using packages *dmetar* [60] and *metafor* [61]. Hedges’ *g* for each for study was calculated using the *esc* [62] in order to minimise the effect of uneven sample sizes [63]. Random-effects models were conducted using the Sidik–Jonkman estimator for heterogeneity with the Hartung–Knapp–Sidik–Jonkman adjustment. These methods were chosen to limit error rates due to expected heterogeneity in studies [64]. Heterogeneity between studies was calculated using Tau^2^ and I^2^ statistics, with interpretation I^2^ as ~25% = low, ~50% = moderate, and ~75% high [65]. Further, to provide additional information about the heterogeneity between studies the prediction interval of studies was also reported [66]. Publication bias was assessed using Eggers’ intercept test and visual analysis of funnel plots [67].

### 2.8. Additional Analyses

Pre-specified subgroup analyses of the effect on intervention type on outcome were conducted. Subgroups were categorised according to intervention type and analysed through random-effects models to assess effect size by intervention and the statistical significance of differences.

## 3. Results

### 3.1. Study Selection

**Figure 1:**
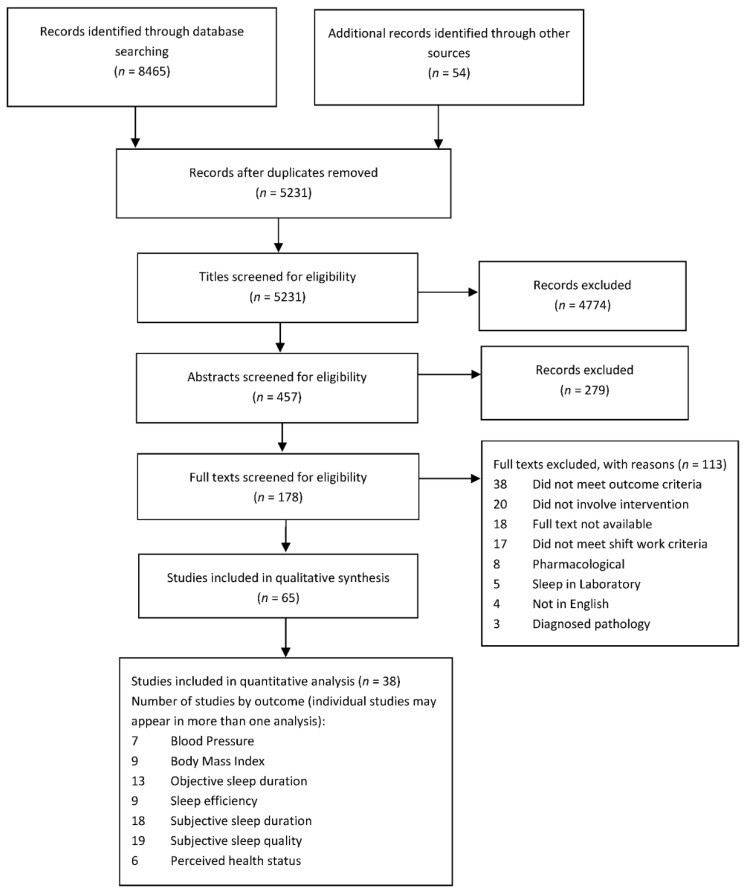
PRISMA flowchart for selection of studies included in review.

The database searches identified 8465 papers. A further 54 papers were identified through hand searching of reference lists and grey literature searches, including using Google Scholar. A PRISMA flowchart for selection of studies, inclusive of updated searches, is depicted in Figure 1. Following duplicate removal, and title and abstract screening, a total of 178 papers remained for full-text review. Full-text reviews were conducted independently by two authors (M.E.C. and A.C.R.). From this review, a further 113 papers were excluded, largely as they did not meet criteria, including absence of a chronic disease risk factor, sleep or perceived health status outcome (*n* = 38), did not involve an intervention (*n* = 20) or the full text was not available (*n* = 18). The total number of studies eligible for inclusion in this review was 65.

### 3.2. Study Characteristics

The mean age of studies was 15.57 years (SD = 10.52), with a range of 0–42 years. The Price index (the percentage of references ≤5 years old) was 21.5% [68,69]. Four common types of non-pharmacological interventions were identified: (1) schedule changes (e.g., switching from backwards rotation to forward rotation or changing length of shifts), (2) behavioural interventions (e.g., sleep education or physical activity program), (3) controlled light exposure (e.g., intermittent bright light or light-blocking glasses), and (4) complementary therapy interventions (e.g., massage or acupuncture). The majority of interventions involved a schedule change (*n* = 30), behavioural change intervention (*n* = 17), or controlled light exposure (*n* = 14). Four studies involved complementary therapy interventions. Table 2 outlines the outcomes reported in each study. Subjective sleep measures were evaluated in 75% of studies (*n* = 49) while 34% (*n* = 22) utilised objective measures of sleep. Biomedical risk factors were evaluated in 39% (*n* = 25) and behavioural risk factors were evaluated in 42% (*n* = 27) of studies. An overview of the data extracted is presented in Table 3, Table 4, Table 5 and Table 6.

### 3.3. Risk of Bias within Studies

Risk of bias assessment ratings varied between 3 and 22 from a possible score of 28, with lower scores reflecting lower quality studies; 90% agreement was achieved between the reviewers. There was considerable variability in risk of bias and quality assessment scores within studies. A full outline of the Downs and Black assessment is provided in Supplementary Material 2.

### 3.4. Participant Characteristics

The studies eligible for qualitative and quantitative analysis included a total of 7806 participants, 56.6% (*n* = 4420) male, 29.1% female (*n* = 2269) and the remaining 14.3% (*n* = 1117) did not report sex of participants. Mean age of participants, where reported, ranged from 23.4 to 52.5 years.

### 3.5. Results of Individual Studies

#### 3.5.1. Schedule Change

As shown in Table 3, change of shift length (38%, *n* = 11) and change of direction of rotation (e.g., change from days to nights instead of nights to days) (31%, *n* = 9) represented the most common interventions. A further 21% (*n* = 6) of interventions utilised increased rest or recovery periods, and 14% (*n* = 4) investigated a change in speed of rotation. One study compared flexible vs. 80 h week limit in medical residents working extended hours.

Interventions involving a change in shift length yielded variable results. Of the eleven studies, 64% (*n* = 7) found that change from an 8 h to a 12 h shift system (e.g., two-shift system) resulted in improvements in subjective sleep parameters, such as increased sleep duration [70,89,91,97] and improved sleep quality [88,93]. Further, a change to 12 h shifts also resulted in decreased blood pressure [91]. However, 18% of studies (*n* = 2) found a negative effect of change to a two-shift system on subjective sleep duration following night shift [77,97]. In addition, a change from 8 to 12 h shifts also resulted in a significant increase in BMI in male clean room workers [99]. One study compared 8, 10 and 12 h shift systems in police officers [71] and found that officers had the longest sleep duration in the 10 h condition, compared to both 8 and 12 h shifts.

Of the studies that changed the direction of shift rotation, 78% (*n* = 7) moved from backward to forward rotation [78,80,83,84,86,90,95]. A further 22% (*n* = 2) investigated a change from forward rotation to backward rotation [72,85]. A change from backward to forward rotation resulted in increased subjective sleep duration [80,83,84,90] and subjective sleep quality [78,83,84,90]. Changing to forward rotation also resulted in improvements in objective sleep efficiency [78]. Further, changing to forward rotation also demonstrated decreases in serum glucose and blood pressure, as well as improved perceived health status [90]. However, the results of change of rotation on blood pressure was not consistent across all studies, with another study finding that a change to forward rotation resulted in increased blood pressure [95]. The change from forward rotation to backward rotation was associated with improved sleep quality and improved perceived health status [85]. In Karlson et al.’s [85] study of rotation change, the change also resulted in increased recovery periods with three days off between schedules. In contrast, another study found that a change from forward to backward rotation was associated with increased sleep difficulties [72]. A change from discontinuous to continuous rotation resulted in improved sleep quality [81]. However, this change was not sustained at 4.5 year follow up [82].

Of the studies that investigated rest periods (*n* = 6), 83% (*n* = 5) involved increased time between shifts and 16% (*n* = 1) introduced a rest period during work hours. Introduction of a scheduled short rest period improved subjective sleep quality [75]. Further, increasing recovery time between evening and morning shifts also improved subjective sleep duration [79]. The introduction of a half-day shift prior to night shift increased objective sleep duration [87,96]. However, both studies were conducted with samples of nurses and over a very short period (3–4 days) and therefore may not be generalisable to other settings or over a longer term. Delaying starting times resulted in improved subjective and objective sleep duration, but decreased sleep quality in steel mill workers [92]. Lastly, increased rest periods resulted in improvements in blood lipids when night shifts were followed by an extra recovery day in nurses and nurses’ aids [74].

Finally, an investigation of length of working week in medical residents found no difference in objective sleep duration between flexible systems and restricted maximum shift hours [73].

#### 3.5.2. Behavioural Interventions

As shown in Table 4, approximately half 44% (*n* = 7) of the interventions that utilised behavioural change methods provided sleep and/or fatigue education sessions, 25% (*n* = 4) investigated prescriptive physical activity interventions and 25% (*n* = 4) targeted health behaviours interventions such as education to improve healthy eating and increasing physical activity. One study investigated a prescriptive bedtime of 10 h prior to shift start time.

In 71% (*n* = 5) of sleep education interventions, there was no significant effect of intervention for objective sleep duration one month [106,113] or four to five months [112] after intervention, subjective sleep duration [112,113,114] or subjective sleep quality [104,113]. However, sleep education utilising individualised cognitive behavioural therapy resulted in improved subjective sleep quality [106]. Further, sleep education resulted in increased subjective sleep duration when education was given both to workers and their immediate family members [105].

Prescriptive physical activity interventions involved supervised training with physical therapist [103,108,109] or weekly consultation with physical therapist via phone [111]. Prescriptive physical activity interventions demonstrated effects in favour of intervention in 75% (*n* = 3) of the studies, namely decreased BMI [111], increased HDL cholesterol [109], and increased subjective sleep duration [103].

Studies that investigated targeted health behaviour interventions utilised weekly team curriculum [101], individualised motivation interviewing [101], increased availability of healthy meals at work [107], a weight loss education session and online resources [110] and tailored health behaviour suggestions based on shifts being worked [115]. Two of these interventions were based on elements of Bandura’s Social Cognitive theory [101,110]. All of the targeted health behavioural interventions found improvements on at least one outcome of interest. Interventions resulted in decreased BMI [110], decreased blood pressure [110], increased physical activity [110,115], increased fruit and vegetable consumption [101] and increased water intake [107]. Further, targeted health behaviour interventions also resulted in significant improvements in sleep quality [115] and perceived health status [101].

Lastly, one study evaluated the effectiveness of prescribing a bedtime ten hours prior to the start time of the following shift. The prescription of bedtime resulted in increased objective sleep duration and increased subjective sleep duration and quality [116]. Further, the sample for this study was small (*n* = 16) but did investigate a range of occupations (manufacturing, customer service, nursing, and public service).

#### 3.5.3. Controlled Light Exposure

As presented in Table 5, of the studies that investigated controlled light exposure interventions (*n* = 14), 50% (*n* = 7) evaluated the effect of intermittent bright light exposure, 36% (*n* = 5) utilised both bright light exposure and light-blocking glasses, while 14% (*n* = 2) investigated dynamic lighting in the workplace. One study investigated the effect of light-blocking glasses alone.

Of the studies that investigated intermittent bright light exposure, an increase in objective sleep duration [123,130], decrease in sleep onset latency [118] and improved subjective sleep quality [128] were observed. However, one study found that bright light exposure resulted in decreased subjective sleep duration [121], while 29% (*n* = 2) found no significant effects associated with bright light exposure [117,120].

In all of the studies that combined bright light exposure and light-blocking glasses, an improvement was found in at least one outcome. In 80% (*n* = 4) of these studies, objective sleep duration increased [119,126,129,130] and in 40% (*n* = 2) studies objective sleep efficiency improved [129,130]. Subjective sleep quality was more variable, with one study finding an increase [124], and another a decrease in subjective sleep quality [129].

Two studies investigated the effect of dynamic lighting in the workplace, both involving nursing populations working within hospitals. Dynamic lighting resulted in no change in sleep parameters [122,127]. However, dynamic lighting was associated with a decrease in perceived health status [127]. Lastly, one study evaluated the effect of light-blocking glasses which resulted in increased objective sleep duration and efficiency [125]. In terms of side effects, participants reported difficultly falling asleep on days off when in the treatment condition [120] and headaches [118,124,127] and eye strain [124]. However, all of these studies report a minority of participants experienced these side effects.

#### 3.5.4. Complementary Interventions

As shown in Table 6, four complementary forms of intervention were included for review. Two studies investigated the effect of massage [131,132], one study investigated the effect of touch therapy [133] and one study investigated the effect of Transcutaneous Electrical Acupoint Stimulation [134]. Half (*n* = 2) of the studies examined blood pressure changes [132,133] and the other half (*n* = 2) examined subjective sleep quality [131,134]. One study investigated objective sleep duration [131]. Two studies [131,134] found significantly improved subjective sleep following intervention. Two of the studies found that control [131] and sham [134] groups also had significantly improved subjective sleep quality. One study found a significant decrease in systolic blood pressure in the control (reading) group but no change in intervention (massage) group [132].

In all four studies, the controls received a work rest break similar to that of the intervention group, but without the treatment. One study found the active control (“sham” acupressure points) group also had significant improvements in subjective sleep quality [134]. Further, a significant improvement in blood pressure in the control (reading and resting) group was found in a separate study [132]. This may suggest that there was a positive effect of a scheduled rest period in the workplace. All four studies were conducted in healthcare personnel, thus the generalisability of these results to other occupations is unknown. Therefore, future studies of complementary therapy in other occupational groups would be of benefit. Complementary therapies may confer benefit for sleep quality in nurses.

### 3.6. Synthesis of Quantitative Results

Eight outcomes were eligible for meta-analysis as there were a minimum of three studies reporting these outcomes. These included systolic blood pressure, diastolic blood pressure, body mass index, objective sleep duration, objective sleep efficiency, subjective sleep duration, subjective sleep quality, and perceived health status. A total of fifty-eight studies were identified as being eligible for inclusion in at least one these meta-analyses. Following the quantitative data procedure detailed in the methods section, nineteen studies had insufficient data and were subsequently excluded from meta-analyses. An outline of included and excluded studies is provided in Table 2.

#### 3.6.1. Blood Pressure

**Figure 2:**
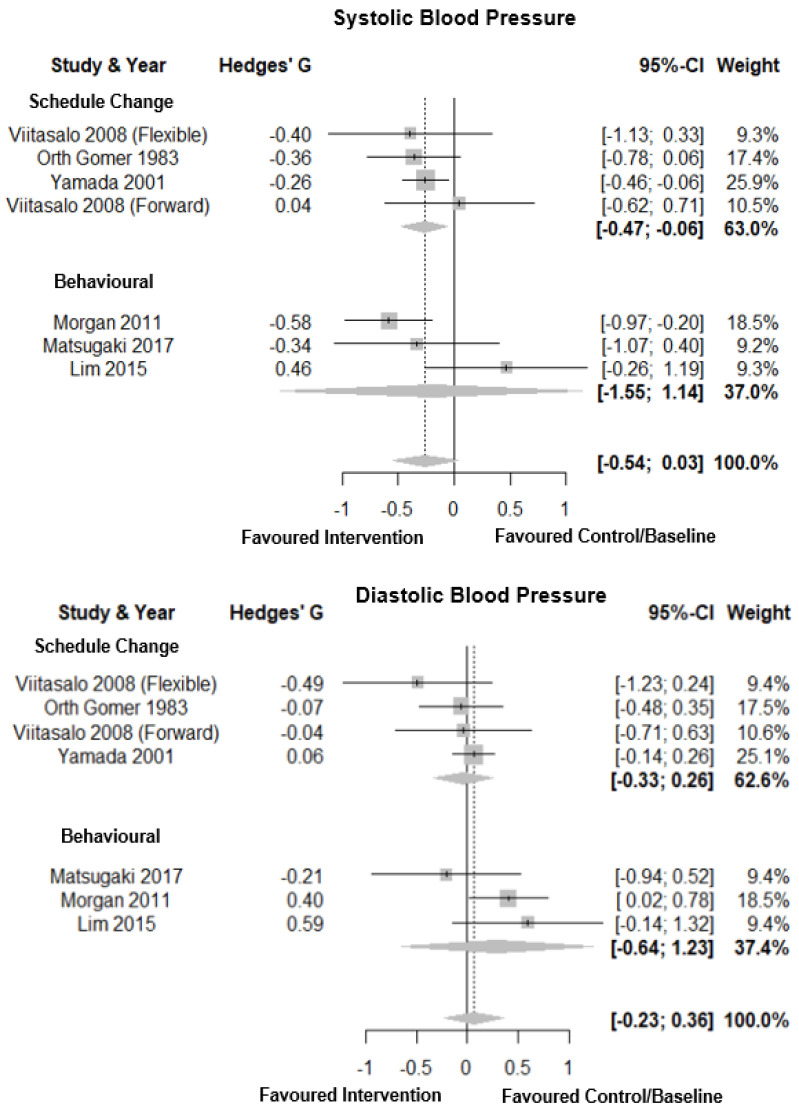
Forest plot for intervention by type on systolic and diastolic blood pressure. Note. Interventions with multiple conditions are denoted within brackets.

**Figure 3:**
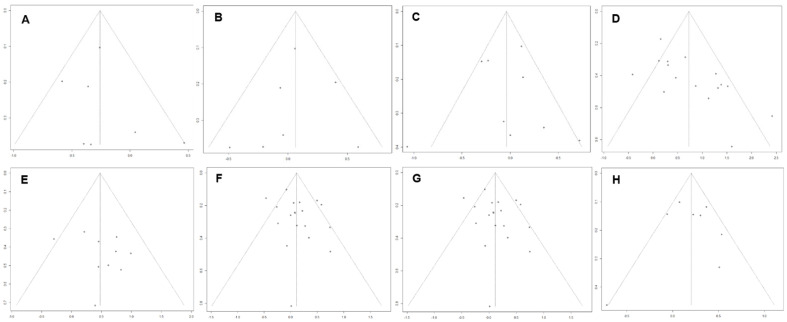
Funnel plots of *x* axis: standardised mean difference, *y* axis: standard error, for studies included in meta-analysis to visually assess publication bias. Panel (**A**) Systolic blood pressure. Panel (**B**) Diastolic blood pressure. Panel (**C**) Body mass index. Panel (**D**) Objective sleep duration. Panel (**E**) Objective sleep efficiency. Panel (**F**) Subjective sleep duration. Panel (**G**) Subjective sleep quality. Panel (**H**) Perceived health status.

Forest plots for blood pressure are shown in Figure 2. Interventions produced a small overall effect on systolic blood pressure (Hedges’ g = 0.26; CI: −0.54, 0.02, *k* = 7), with low heterogeneity (τ^2^ = 0.07 and I^2^ = 21.2%) and a prediction interval of −0.96; 0.44. Eggers’ test showed no evidence of publication bias (*p* = 0.58, Figure 3A). Subgroup analysis showed no significant difference between intervention types for effect on systolic blood pressure, with a small negative pooled effect for both schedule change (Hedges’ g = −0.26; CI: −0.47, −0.06, *k* = 4, I^2^ = 0.0%) and behavioural interventions (Hedges’ g = −0.20; CI: −1.55, −1.14, *k* = 3, I^2^ = 67.9%). Interventions produced a very small effect on diastolic blood pressure (Hedges’ g = 0.06; CI: −0.23, 0.36, *k* = 7), with low-moderate heterogeneity (τ^2^ = 0.06 and I^2^ = 26.3%) and a prediction interval of −0.67; 0.80. There was no evidence of publication bias (*p* = 0.78, Figure 3B). Subgroup analysis showed no significant differences between intervention types with behavioural interventions showing a medium combined effect size (Hedges’ g = 0.29; CI: −0.65, −1.23, *k* = 3, I^2^ = 26.4%) and a small negative total pooled effect of schedule change (Hedges’ g = −0.04; CI: −0.33, 0.26, *k* = 4, I^2^ = 0.0%).

#### 3.6.2. Body Mass Index

**Figure 4:**
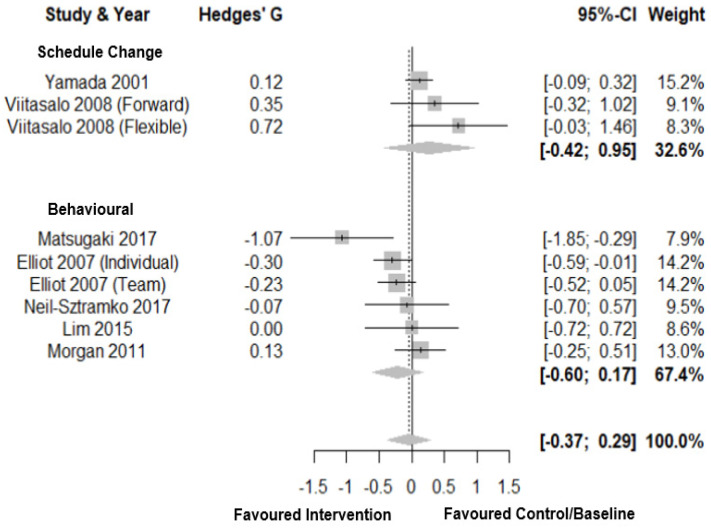
Forest plot for intervention by type on body mass index. Note. Interventions with multiple conditions are denoted within brackets.

Forest plots for body mass index are shown in Figure 4. Interventions produced a small effect on BMI (Hedges’ g = −0.04; CI: −0.37, 0.29, *k* = 9), with moderate heterogeneity (τ^2^ = 0.15 and I^2^ = 59.5%) and a prediction interval of −1.02; 0.94. Subgroup analysis showed a significant difference between intervention type (*p* = 0.03) with behavioural interventions producing a small effect in favour of the intervention (Hedges’ g = −0.21; CI: −0.59, 0.17) and schedule change producing a small effect against the intervention (Hedges’ g 0.27: CI: −0.42, 0.95). There was no evidence of publication bias (*p* = 0.94, Figure 3C).

#### 3.6.3. Objective Sleep Duration

**Figure 5:**
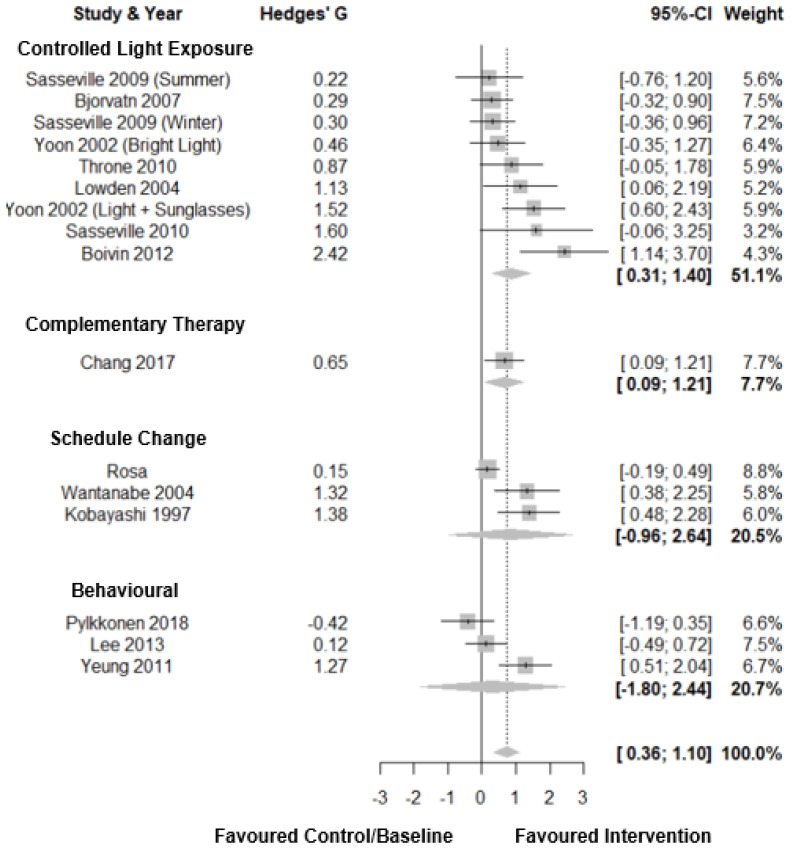
Forest plot for intervention by type on objective sleep duration. Note. Interventions with multiple conditions are denoted within brackets.

Forest plots for objective sleep duration are shown in Figure 5. There was a medium to large pooled effect size (Hedges’ g = 0.73; CI: 0.36, 1.10, *k* = 16), with moderate heterogeneity (τ^2^ 0.36 and I^2^ = 62.4%) with a prediction interval of −0.61; 2.01. Subgroup analysis showed a large pooled effect for controlled light exposure (Hedges’ g = 0.86; CI: 0.31, 1.41, *k* = 9) and schedule change interventions (Hedges’ g = 0.84; CI: −0.96, 2.64, *k* = 3), There was a medium pooled effect size for complementary therapies (Hedges’ g = 0.65; CI: 0.09, 1.21, *k* = 1), and a small pooled effect for behavioural interventions (Hedges’ g = 0.32; CI: −1.80, 2.44, *k* = 3). However, there was no significant difference by intervention type (*p* = 0.77). Eggers’ test indicated possible publication bias (*p* = 0.005, Figure 3D).

#### 3.6.4. Sleep Efficiency

**Figure 6:**
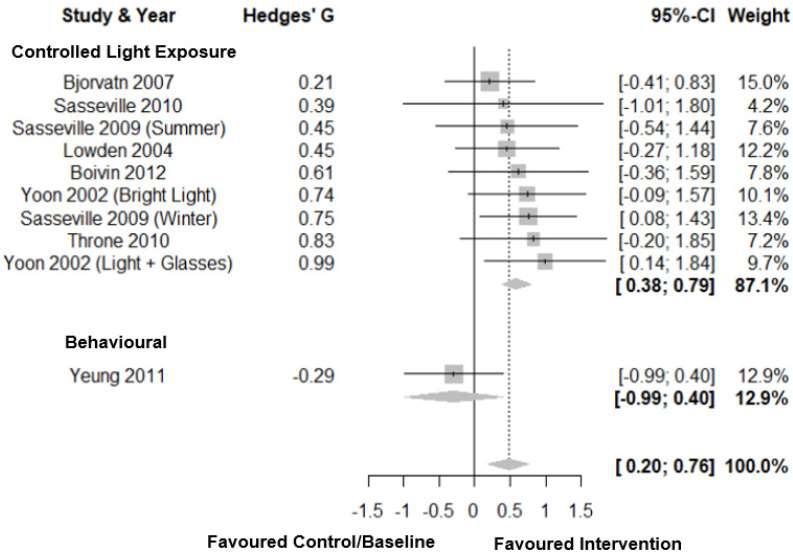
Forest plot intervention by type on sleep efficiency. Note. Interventions with multiple conditions are denoted within brackets.

Forest plots for sleep efficiency are shown in Figure 6. For sleep efficiency there was a medium total pooled effect size (Hedges’ g = 0.48; CI: 0.20, 0.76, *k* = 10) with low heterogeneity (τ^2^ = 0.06 and I^2^ = 0.0%) and a prediction interval of −0.16; 1.11. Subgroup analysis showed a significant difference between intervention types (*p* = 0.02) with a medium total pooled effect size for controlled light exposure on sleep efficiency (Hedges’ g = 0.59; CI: 0.38, 0.79, *k* = 9) and a small negative pooled effect size for behavioural interventions (Hedges’ g = −0.29; CI: −0.99, 0.40, *k* = 1). Eggers’ test of the intercept indicated no evidence of publication bias (*p* = 0.35, Figure 3E).

#### 3.6.5. Subjective Sleep Duration

**Figure 7:**
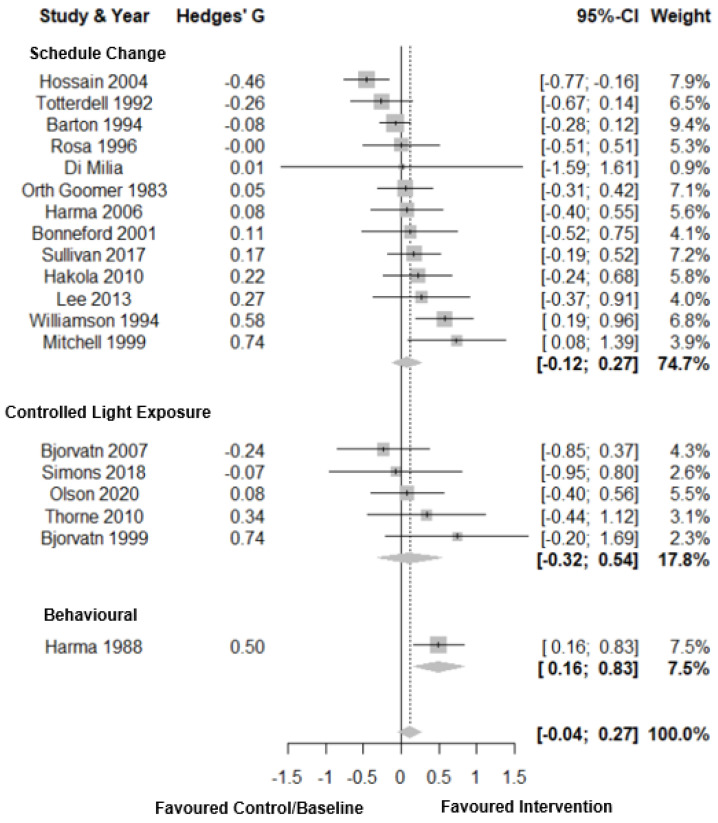
Forest plot for intervention by type on subjective sleep duration.

Forest plots for subjective sleep duration are shown in Figure 7. For subjective sleep duration, there was a small total pooled effect size (Hedges’ g = 0.11; CI: −0.04, 0.27, *k* = 19) with moderate heterogeneity (τ^2^ = 0.06 and I^2^ = 52.9%) and a prediction interval of −0.43; 0.66. Subgroup analysis showed no significant difference between intervention types. For behavioural change there was a medium total pooled effect size (Hedges’ g = 0.50; CI: 0.16, 0.83, *k* = 1). There was a small total pooled effect size for both controlled light exposure (Hedges’ g = 0.11; CI: −0.32, 0.54, *k* = 13) and schedule change interventions (Hedges’ g = 0.08; CI: −0.12, 0.27, *k* = 13). There was no evidence of publication bias (*p* = 0.20, Figure 3F).

#### 3.6.6. Subjective Sleep Quality

**Figure 8:**
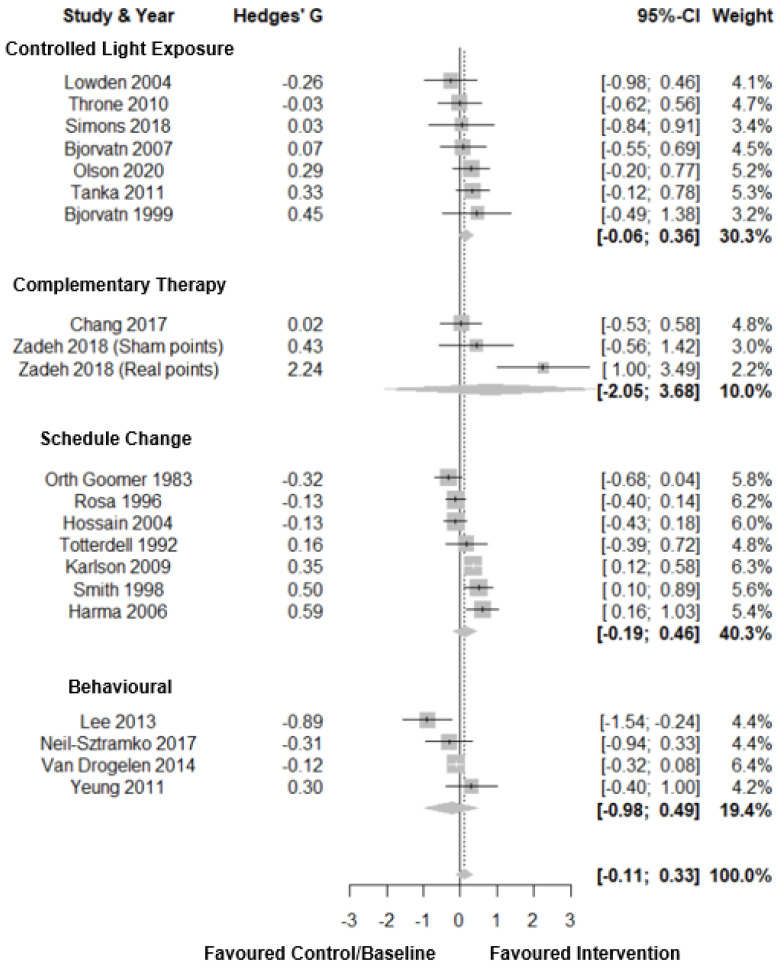
Forest plot of interventions by type on subjective sleep quality. Note. Interventions with multiple conditions are denoted within brackets.

Forest plots for subjective sleep quality are shown in Figure 8. For subjective sleep quality, there was a small total pooled effect size (Hedges’ g = 0.11; CI: −0.11, 0.33, *k* = 21) with moderate heterogeneity (τ^2^ = 0.21 andI^2^ = 63.8%) and a prediction interval of −0.89; 1.10. Subgroup analysis showed no significant difference between intervention types. For complementary therapies there was a large total pooled effect size (Hedges’ g = 0.81; CI: −2.05, 3.68, *k* = 3). There was a small total pooled effect size for both controlled light exposure (Hedges’ g = 0.15; CI: −0.06, 0.36, *k* = 7) and schedule change interventions (Hedges’ g = 0.13; CI: −0.19, 0.46, *k* = 7). There was a small negative total pooled effect for behavioural interventions (Hedges’ g = −0.24; CI: −0.98, 0.49, *k* 4). There was no evidence of publication bias (*p* = 0.34, Figure 3G).

#### 3.6.7. Perceived Health Status

**Figure 9:**
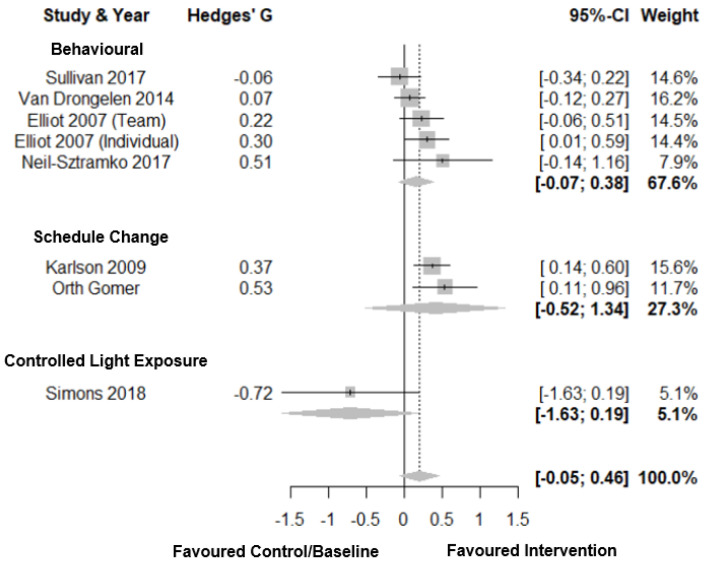
Forest plot for interventions by type on perceived health status. Note. Interventions with multiple conditions are denoted within brackets.

Forest plots for perceived health status are shown in Figure 9. For perceived health status, there was a small total pooled effect size (Hedges’ g = 0.20; CI: −0.05, 0.46, *k* = 8) with moderate heterogeneity (τ^2^ = 0.08 and I^2^ = 52.0%) and a prediction interval of −0.56; 0.96. Subgroup analysis showed no significant difference between intervention types. There was a small to medium total pooled effect size for schedule change (Hedges’ g = 0.41; CI: −0.52, 1.34, *k* = 2) and small total pooled effect for behavioural interventions (Hedges’ g = 0.16; CI: −0.07, 0.38, *k* = 5). There was a large negative total pooled effect for controlled light exposure (Hedges’ g = −0.72; CI: −1.63, 0.19, *k* = 1). There was no evidence of publication bias (*p* = 0.96, Figure 3H).

## 4. Discussion

### 4.1. Summary of Evidence

The present review provides the first comprehensive qualitative and quantitative analysis of the scope and effectiveness of non-pharmacological interventions for chronic disease risk factors, sleep outcomes and perceived health status in shift workers. The present review showed that schedule change, behavioural change, controlled light exposure and complementary therapy interventions have been used to improve chronic disease risk factors and sleep in shift workers.

### 4.2. Meta-Analytic Findings

In considering biomedical risk factors, only the outcomes of blood pressure and BMI had sufficient studies to be included for meta-analysis to date. For behavioural risk factors, there were insufficient studies for meta-analysis across all risk factors. Both objective sleep measures and subjective sleep measures had sufficient literature to allow for meta-analysis. There were also sufficient studies examining perceived health status for meta-analysis. Heterogeneity was common, likely reflecting the varied methods of intervention utilised and differences between occupational settings.

In studies with sufficient data for meta-analysis, there appear to be small favourable effects of interventions on biomedical risk factors, being blood pressure (both systolic and diastolic) and BMI. Further, subgroup analysis showed a significant difference of effect by intervention type, with behavioural interventions resulting in favourable effects. For objective sleep measures, there were large favourable effects of intervention on sleep duration and medium effects on sleep efficiency. In addition, subgroup analysis showed that controlled light exposure had a significant effect on sleep efficiency compared to behavioural interventions. However, subjective measures of sleep and perceived health status only showed small effects of intervention, with no differences between intervention types.

### 4.3. Qualitative Findings

The present review indicates that interventions aimed at improving sleep in shift workers are well represented, albeit diverse in their approaches and measurement of other chronic disease risk factors. This is unsurprising given the immense evidence of the negative sleep effects associated with shift work [1,2,3,28,29,30]. However, consistent measures of biomedical and behavioural risk factors were present in relatively few studies. This indicates a need for future research which considers risk factors associated with chronic disease, beyond sleep outcomes, if we are to inform a strong evidence base for early (pre-disease) intervention in shift workers.

Findings differed across outcome and intervention type and therefore, it is not possible to suggest that any one type of intervention may be best to improve overall chronic disease risks for shift workers. This is not surprising—while shift work may be a common feature for these workers, individual differences in occupation, sociodemographic characteristics, job demands, and health status likely call for unique interventions. Broadly, objective sleep measures were improved by controlled light exposure including intermittent bright light [123,130], bright light and light-blocking goggles [119,126,129,130], light-blocking goggles alone [125], schedule change which increased recovery periods [87,92,96] and prescriptive sleep scheduling [116]. Subjective measures of sleep were improved by a change to 12 h shifts (e.g., two-shift system) [70,88,89,91,93,97], a change to forward rotation schedules [78,80,83,90], increased rest or recovery periods [75,79], individualised behavioural intervention aimed at improving sleep and fatigue [106,115], and some complementary therapies (e.g., electrical acupoint stimulation and aromatherapy massage) [131,134].

Of those biomedical risk factors reported, most did not change significantly, except for a decrease in blood pressure associated with change from backward to forward rotation [90], a change to 12 h shifts [91] and a behavioural intervention targeting weight loss and healthy eating [110]. Further, cholesterol showed some improvements in schedules that increased recovery periods [74] and a prescribed physical activity program [109]. BMI significantly decreased in behavioural interventions targeting weight loss through healthy eating and increased physical activity [110] and prescribed physical activity intervention [111].

When evaluating behavioural risk factors, an increase in physical activity was associated with prescribed physical activity intervention [111], interventions based on Social Cognitive theory [110] and personalised behavioural interventions [115]. The only study reporting a significant difference in nutritional intake was a behavioural intervention based on Social Cognitive theory [101]. Finally, perceived health status was improved with behavioural intervention based on Social Cognitive theory [101], change to a slower rotation schedule [76] and change to slow backward rotation schedule [85].

Schedule change interventions were commonly used. This is largely unsurprising, as an extensive body of literature has shown that schedule characteristics are associated with health and safety outcomes [135,136,137,138,139,140]. Results of these interventions favoured two shift changes, a forward-rotating shift system and increased length of recovery periods. While it is positive that schedule changes can benefit individual worker health, one of the major limitations of schedule changes is that they require management to be actively involved and require change at an organisational level, which is not always simple. Further, comparison of interventions involving schedule changes are somewhat hampered by heterogeneity in the shift systems being worked before workers underwent respective interventions. Studies utilised differing shift designs at baseline, therefore further complicating the ability to compare across all schedule change interventions. Thus, while changing schedules to optimal timing for workers is likely beneficial for both safety and health outcomes, additional individual interventions may be needed for workers.

Behavioural interventions resulted in some improvements in sleep and other health indicators. Importantly, behavioural interventions that tailored the information provided to workers were more likely to result in positive changes. This is consistent with previous studies comparing individualised to non-individualised interventions for health behaviours [141,142]. An example is the study by Van Drongelen et al. [115], in which a control group received general information regarding fatigue and health behaviours, while the intervention group received personalised information. The findings suggest that without tailoring educational resources, providing information about fatigue, sleep behaviours or healthy diet may not be effective in a shift-working population. It is possible that these findings can be attributed to the complex nature of behavioural change. Shift workers may not know that they personally are at risk of poorer health outcomes and thus may not interpret the information provided as relevant to them. Thus, the use of a behavioural change framework for future interventions may be effective. Further, where behavioural interventions were informed by an existing behavioural model, studies yielded significant improvements on various outcomes indicating the possible utility of adapting existing behavioural models for use in shift-working populations to improve chronic disease risk factors.

Qualitative analysis indicates that controlled light therapy was beneficial for shift workers, particularly for improved objective sleep parameters. This was supported by meta-analysis. However, the strength and timing of bright light varied greatly between studies. Unfortunately, there were not enough studies to conduct subgroup analysis by light timing or strength. Consequently, there remains limited evidence to date to establish the most suitable timing or intensity of light administration to provide clear guidelines for shift workers. Additionally, the available studies were largely conducted on nursing populations or those working offshore, therefore limiting our understanding of controlled light exposure in other occupational settings. The overrepresentation of these occupations may simply indicate that the use of controlled light may be more practical in these settings where nurses have common staff facilitates (e.g., break room) and offshore industrial workers living on location. Feasibility for other shift-working populations is unclear and warrants further consideration.

It is also important to note that controlled light appeared to be associated with some negative side effects (e.g., headaches or eyestrain) [118,120,124,127], decreased subjective sleep quality [129] and deterioration in perceived health status [127]. Future studies should include sufficient measures of possible negative side effects when conducting controlled light interventions. It appears that an important balance will need to be found between use of light therapy and well-being from the individual worker’s perspective. Taking an individualised approach to light therapy such as adjusting the timing and strength of light exposure according to shift schedule [143] may be an option; however, future studies should first consider feasibility (and sustainability) of personalised approaches in a robust, experimental manner.

Complementary therapies, whilst limited in number, showed interesting results for health risk indicators in shift workers. Both studies that investigated subjective sleep quality found a significant positive effect of intervention on sleep quality. However, a limitation of the literature on complementary therapies in shift workers is that all were conducted within healthcare populations, limiting the generalisability. It is unclear whether the same benefits would be observed in other occupations as no complementary interventions used in other occupational groups were identified. While the literature here is limited, it is feasible that complementary therapies may afford benefits for sleep in shift workers. Future studies could consider shift workers from other occupations to identify specific worker populations for whom complementary therapies confer greatest benefit.

The present systematic review is strengthened by an integration of qualitative and quantitative analysis. Further, use of chronic disease criteria in combination with sleep and perceived health status allowed for a robust understanding of interventions targeting improvements to chronic disease risk factors in shift workers. Beyond this, for meta-analytic data, the present review evaluated effect sizes by outcome, and also by intervention type. This allowed for quantitative understanding of the impact of type of intervention on various outcomes. Finally, the present quantitative analysis was based on the combination of effect sizes when sleep outcomes were reported by shift, allowing for comparison of total sleep duration and quality across all available studies. This technique overcomes some reporting barriers which make qualitative comparisons difficult, while also allowing for a consistent measure of the impact of intervention on total sleep time.

### 4.4. Limitations

A substantial number of studies could not be included in meta-analyses due to insufficient reporting or data that were not compatible with measures of standardised mean difference (see Table 2). Therefore, while these meta-analytic outcomes provide preliminary understanding of the effect of interventions on some important health measures in shift workers, it is necessary to acknowledge not all studies have been included. This highlights the importance of future data sharing and open science practices to allow for accurate evaluation of intervention studies.

The review is limited by the minimal investigation of biomedical and behavioural risk factors associated with downstream chronic disease in shift work interventions. Given that each of these risk factors is known to contribute to the development of various chronic disease, it is important that such risk factors (beyond sleep outcomes) are targeted within a shift-working population. Another limitation within the current studies is the relatively limited studies that utilised an individualised design. Personalisation of behavioural interventions [141,142] and controlled light exposure [143] have been shown to have positive effects for individuals and should be considered for future intervention studies.

Quality assessment and risk of bias indicated significant variability in reporting, external validity, study bias and possible confounding. The average estimates of these scores were impacted by studies that scored particularly low on the assessment scale [86,105]. Beyond this, a difference among studies is not unexpected as the review included both randomised trials and observational studies and did not limit studies to recent publications. Nonetheless, it is crucial that future studies and subsequent publications aim to limit bias and confounding wherever possible and ensure greater transparency with reporting in publications.

Another limitation of the studies within the present review is that many interventions were only reported in one occupational group, and thus generalisability to other occupational groups may be limited. This highlights a pressing need for expanded intervention studies in shift-working populations in the future in order to address the known long-term chronic disease outcomes associated with this work pattern. Further, studies differed in both baseline characteristics and intervention types making meaningful comparison challenging. It will be useful for future studies to consider these existing interventions when designing new studies in order to allow for comparison with existing literature. Lastly, none of the current interventions address all the chronic disease risk factors outlined, therefore limiting our ability to compare current interventions on all risk factors. Future studies may consider including additional outcomes to allow for analysis of the effect of study across multiple chronic disease risk factors.

Consequently, it is acknowledged that shift work is associated with increased risk of occupational injury [46,144,145,146,147], psychological pathology [148,149,150,151], and poor fertility outcomes [152,153,154]. However, safety, psychological and fertility outcomes present additional paradigms which, while very important, were beyond the scope of this review.

## 5. Conclusions

The findings of this systematic review and meta-analysis suggest that some chronic disease risk factors, particularly sleep outcomes, can be improved with interventions. Objective sleep outcomes are improved with a medium to large effect, with controlled light exposure and schedule change producing large favourable effects. Subjective sleep duration is improved with a small effect size, with behavioural interventions contributing the largest positive effect. Subjective sleep quality is also improved, with a small effect size, with complementary therapies producing the largest effect. Qualitative analysis suggests that two-shift, forward-rotating schedules, increased recovery periods, tailored behavioural interventions, interventions based on existing behavioural theoretical frameworks and intermittent bright light and light-blocking goggles, improve some chronic disease risk factors, sleep and perceived health status in shift workers. The limited volume of interventions targeting biomedical and behavioural risk factors in shift workers indicates a need for further studies within this area.

Future studies should consider the efficacy of existing interventions, as analysed in this review. The implementation of promising interventions such as two-shift schedules, increased rest periods, bright light exposure combined with light-blocking goggles, and tailored behavioural interventions in different occupational groups and across larger samples would provide additional understanding of the feasibility and effectiveness of these interventions for all shift workers. Importantly, future studies should consider targeting various chronic disease outcomes, aim to take all practical steps to limit bias and confounding, and to report as many study details as possible in order to assist in the further development and enhancement of interventions to improve chronic disease, sleep and health outcomes in shift workers.

## Figures and Tables

**Table 1:** Associations between common chronic diseases and chronic disease risk factors (biomedical and behavioural).

	Biomedical Risk Factors				Behavioural Risk Factors			
Common Chronic Disease	High Blood Pressure	Obesity	Dyslipidaemia	Abnormal Glucose	Tobacco Smoking	Insufficient Physical Activity	Excessive Alcohol Consumption	Poor Nutritional Intake
Cardiovascular disease							-	
Stroke				-				-
Type 2 diabetes	-		-				-	
Colorectal cancer	-		-	-		-		
Osteoporosis	-	-	-	-				
Breast cancer (in females)	-		-	-	-	-		-
Chronic kidney disease			-	-		-	-	-

Note. Adapted with permission from AIHW material [31]. Shaded square with dot, indicates a direct association between risk factor and chronic disease. Non-shaded squares indicate insufficient evidence of direct relationship; however, an indirect relationship may exist.

**Table 2:** Outcomes reported by each study, with shaded boxes indicating inclusion in meta-analysis.

		Biomedical Risk Factors				Behavioural Risk Factors				Sleep				Global Health
Study, Year	Intervention Type	Blood Pressure	Body Mass Index	Dyslipidemia (d)	Glucose (d)	Tobacco Smoking (d)	Physical Activity (d)	Alcohol Intake (d)	Nutritional Intake (d)	Objective Sleep Duration	Objective Sleep Efficiency	Subjective Sleep Length	Subjective Sleep Quality	Perceived Health Status
**Akerstedt, 1978**	schedule											(b)		
**Amendola, 2011**	schedule											(b)	(b)	
**Arora, 2007**	behaviour									(c)				
**Barton, 1994**	schedule													
**Basner 2019**	schedule									(c)			(c)	
**Bjorvatn, 1999**	light											(c)	(c)	
**Bjorvatn, 2007**	light													
**Bøggild, 2001**	schedule			(b)		(b)	(b)	(b)						
**Boivin, 2012**	light													
**Bonneford, 2001**	schedule												(b)	
**Budnick, 1995**	light											(b)	(b)	
**Chang, 2015**	complementary													
**Costa 1993**	light											(b)		
**Czeisler, 1982**	schedule												(b)	(b)
**Di Milia, 1998**	schedule											(b)		
**Elliot, 2007**	behaviour						(b)							
**Fazeli, 2020**	complementary	(b)												
**Hakola, 2002**	schedule									(b)	(b)	(b)	(b)	
**Hakola, 2010**	schedule													
**Harma, 2006**	schedule									(b)	(b)			
**Härmä, 1988**	behaviour												(b)	
**Holbrook, 1994**	behaviour												(b)	
**Hornberger, 1995**	schedule												(b)	
**Hornberger, 1998**	schedule												(c)	
**Hossain, 2004**	schedule									(c)	(c)			(a)
**Jensen, 2016**	light									(b)	(b)	(b)	(b)	
**Karhula, 2020**	schedule					(a)	(a)	(a)				(b)	(b)	(a)
**Karlson, 2009**	schedule													
**Kerin, 2005**	behaviour											(b)		
**Knauth, 1998**	schedule											(b)	(b)	
**Kobayashi, 1997**	schedule													
**Lee, 2014**	behaviour													
**Leedo, 2017**	behaviour		(a)											
**Lim, 2015**	behaviour													
**Lowden, 2004**	light											(b)		
**Lowden, 1998**	schedule									(b)	(b)	(b)	(b)	(b)
**MacKinnon, 2010**	behaviour		(c)				(c)		(c)					(c)
**Matsugaki, 2017**	behaviour													
**McElligott, 2003**	complementary	(b)												
**Mitchell, 2000**	schedule												(b)	
**Morgan, 2011**	behaviour							(b)						
**Neil-Sztramko, 2017**	behaviour													
**Olson, 2020**	light						(a)							
**Orth Gomer, 1983**	schedule			(b)	(b)									
**Peacock, 1983**	schedule											(b)	(b)	
**Pylkkönen, 2018**	behaviour											(a)	(a)	
**Rosa, 1996**	schedule													
**Sasseville, 2009**	light													
**Sasseville, 2010**	light													
**Simons, 2018**	light													
**Smith, 1998**	schedule											(b)	(b)	
**Smith-Coggins, 1997**	behaviour									(b)		(b)	(b)	
**Sullivan, 2017**	behaviour													
**Tanaka, 2011**	light													
**Thorne, 2010**	light		(a)											
**Totterdell, 1992**	schedule													
**van Drongelen, 2014**	behaviour								(b)			(b)		
**Viitasalon, 2008**	schedule						(b)	(b)	(b)				(a)	
**Watanabe, 2004**	schedule													
**Williamson, 1994**	schedule												(b)	
**Williamson, 1986**	schedule					(b)			(b)			(b)	(b)	
**Yamada, 2001**	schedule													
**Yeung, 2011**	behaviour											(b)		
**Yoon, 2002**	light											(a)		
**Zadeh, 2018**	complementary													

Note. Shaded squares indicate studies included in meta-analysis for corresponding outcome. (a) Outcome was not reported at follow up or was only used as a co-variate, (b) insufficient information reported for means and/or standard deviations to be calculated, (c) not eligible for inclusion in meta-analysis (follow-up study or extended working hours), and (d) meta-analysis not conducted due to insufficient studies.

**Table 3:** Studies investigating schedule change interventions.

Author Year	Sample Size	Sample Characteristics (Sex, Mean Age)	Occupation	Type of Shift Work at Baseline	Intervention Detail	Intervention Duration	Outcome Measure (Measurement Used)	Results
Akerstedt et al., 1978 [70]	361	Not reported	Steel manufacturing employees	Rotating shift work	(I1) change from 3/4 shift to 2 shift (*n* = 69) (I2) change from 4 to 3 shift (*n* = 41) (C1) 2 shift no change (*n* = 16) (C2) 3 shift no change (*n* = 73) (C3) 4 shift no change (*n* = 77)	12 months	Subjective sleep duration (questionnaire)	(I1) Increased subjective sleep duration (I2) Decrease in subjective sleep duration
Amendola et al., 2011 [71]	226	M = 174 F = 52 Age not reported	Police officers	Not reported	Randomly assigned to 8, 10 or 12 h shifts	6 months	Subjective sleep duration (aleep diary) Subjective sleep quality (sleep diary)	Officers in the 10 h condition averaged more hours of sleep than officers in the 8 and 12 h shifts
Barton et al., 1994 [72]	293	M = 271 F = 22 30.2 years	Car manufacturing employees	Rotating shift work	Change from an 8 h forward rotation to an 8 h backward system	6 months	Cigarette consumption (questionnaire) Alcohol consumption (questionnaire) Subjective sleep duration (sleep diary) Subjective sleep quality (sleep diary)	Increased sleep difficulties between afternoon shifts
Basner et al., 2019 [73]	398	M = 203 F = 195 27.9 years	Medical interns	Rotating shift work	(i) Standard 2011 duty hour policies (ii) Flexible policies—80 h work week without limits on shift duration or mandatory time off between shifts	14 days	Objective sleep duration (actigraphy)	No significant difference in objective sleep duration
Bøggild et al., 2001 [74]	101	Sex not reported Median age: Intervention = 34.5 Control = 42.0	Nurses and nurses′ aids	Rotating and permanent evening or night shifts	Ergonomic shift design—more regular, more weekends off and maximum of 3 to 4 consecutive night shifts followed by extra day off	6 months	Cholesterol (HDL-C, LDL-C) Cigarette consumption (Standard Shiftwork Index) Physical activity (Standard Shiftwork Index) Alcohol consumption (Standard Shiftwork Index)	HDL-C level increased LDL-C and the total HDL-C cholesterol ratio decreased
Bonnefond et al., 2001 [75]	12	M = 12 F = 0 37.0 years	Electric power plant employees	Rotating shift work	One short rest period approx. 1 h, during nightshift whenever possible	1 year	Subjective sleep duration (sleep diary) Subjective sleep quality (sleep diary)	Improved subjective sleep quality
Czeisler et al., 1982 [76]	85	M = 85 F = 0 31.4 years	Minerals and chemical corporation plant employees	Rotating shift work	Intervention: 52 others rotated shifts by phase delay once every 21 days Control: 33 workers continued to change shifts each week	9 months	Subjective sleep quality (sleep–wake questionnaire) Perceived health status (questionnaire)	Improved perceived health status
Di Milia et al., 1998 [77]	3	M = 3 F = 0 Ages 34, 27, 30 years	Electricians in coal mine	Rotating shift work	Change from 8 to 12 h system	11 months	Subjective sleep duration (sleep diary)	Decrease in subjective sleep duration on night shift
Hakola and Harma, 2002 [78]	16	M = 16 F = 0 42.0 years	Steel industry employees	Rotating shift work	A continuous three-shift schedule was changed from a slow backward-rotating to a fast forward-rotating system	1 year	Objective sleep duration (actigraphy) Objective sleep efficiency (actigraphy) Subjective sleep duration (sleep log) Subjective sleep quality (sleep log)	Improved objective sleep efficiency Improved subjective sleep quality after morning shift
Hakola et al., 2010 [79]	75	M = 3 F = 72 46.0 years	Nurses	Rotating shift work	Increased recovery time between evening and morning shifts	1 year	Subjective sleep duration (Modified Standard Shiftwork Index)	Increased subjective sleep duration
Harma et al., 2006 [80]	140	M = 140 F = 0 45- Control = 36.0 45- Intervention = 36.0 45+ Control = 50.0 45+ Intervention = 52.0	Maintenance employees	Rotating shift work	Change from backward-rotating three-shift system to very quickly forward-rotating shift system was developed	6 months	Objective sleep (actigraphy) Subjective sleep duration (sleep diary) Subjective sleep quality (sleep diary)	Increased subjective sleep duration following night shift
Hornberger et al., 1995 [81]	260	M = 260 F = 0 Group B = 38.2 years Group E = 38.9 years	Chemical industry employees	Rotating shift work	Change from discontinuous to continuous shift system. Change to faster rotation with shorter working days	7–9 months	Subjective sleep quality (questionnaire)	2 groups showed improved subjective sleep quality 1 group showed decreased subjective sleep quality
Hornberger et al., 1998 [82] Follow up of study Hornberger et al., 1995	50	M = 50 F = 0 32.8 years	Chemical industry employees	Rotating shift work	Change from discontinuous to continuous shift system. Change to faster rotation with shorter working days	4.5 year follow up	Subjective sleep quality (questionnaire)	No change
Hossain et al., 2004 [83]	58	M = 58 F = 0 40.3 years	Mine workers	Rotating shift work	Change from a backward-rotating 8 h to a forward-rotating 10 h shift schedule	1 month	Subjective sleep quality (shift work and you) Subjective sleep duration (shift work and you) Perceived health status (shift work and you)	Increased subjective sleep duration on day shift schedule Improved subjective sleep quality on night shift and days off
Karhula et al., 2020 [84]	1487	M = 70 F = 1417 Control = 52.3 years Intervention = 52.5 years	Social and healthcare employees (nurses, nurse assistants, social workers)	Rotating shift work	Ergonomic roster: Change from backward to forward rotation. Max of 50 h worked per week. Night shifts followed by at least two days off. Maximum shift shift 10 h. Increased recovery time between shifts.	5–6 years	Subjective sleep duration (self-report average 24 h sleep duration) Subjective sleep quality (survey for past 4 weeks) Smoking (Yes/No) Physical activity (self-report hours per week) Alcohol intake (questionnaire) Perceived health status (“How is your health compared to someone else your age?”)	Improved subjective sleep quality Increases in intervention workers reporting long sleep (>9 h) Improved perceived health status
Karlson and Eek, 2009 [85]	118	M = 98 F = 20 44.6 years	Manufacturing plant employees	Rotating shift work	Change from fast forward rotation to a slower backward rotation with 3 days on a given shift followed by 3 days off	15 months	Subjective sleep quality (Karolinska Sleep Questionnaire) Perceived health status (self-rated health question)	Improved sleep quality Improved perceived health status
Knauth and Hornberger, 1998 [86]	90	Sex not reported E1—35.6 years C1—39.8 years E2—34.1 years C2—35.8 years	Steel industry employees	Rotating shift work	(El) changed from a discontinuous weekly backward-rotating to a quicker forward-rotating shift system (E2) first worked in a weekly backward-rotating and then in a quicker forward-rotating shift system	10 months	Subjective sleep duration (questionnaire) Subjective sleep quality (questionnaire)	No significant differences
Kobayashi et al., 1997 [87]	12	M = 0 F = 12 24.8 years	Nurses	Rotating shift work	Change from full-day shift to half-day shift before night shift	3 days	Objective sleep duration activity (actigraphy)	Increased objective sleep duration
Lowden et al., 1998 [88]	34	M = 30 F = 4 34.0 years	Chemical plant employees	Rotating shift workers	Change from rotating 3 shift (8 h) to a 2 shift (12 h) schedule	10 months	Objective sleep duration (actigraphy) Objective sleep efficiency (actigraphy) Subjective sleep duration (Karolinska sleep diary) Subjective sleep quality (Karolinska sleep diary) Perceived health status (questionnaire)	Improved subjective sleep quality
Mitchell et al., 1999 [89]	27	M = 27 F = 0 8 h = 43.8 years 12 h = 44.3 years	Power station employees	Rotating shift work	Change from 8 to 12 h shifts	10 months	Subjective sleep duration (sleep dairy) Subjective sleep quality (sleep diary)	Increased subjective sleep duration Improved subjective sleep quality
Orth Goomer, 1983 [90]	45	M = 45 F = 0 Group 1 = 30.4 years Group 2 = 30.8 years	Police officers	Rotating shift work	Change from backward rotation to forward rotation	8 week crossover	Blood pressure Cholesterol (not reported) Serum glucose Subjective sleep duration (post-sleep assessment) Subjective sleep quality (post-sleep assessment) Tobacco smoking (questionnaire) Perceived health status (questionnaire)	Systolic blood pressure decreased Lowered serum glucose Increased subjective sleep duration for night sleeps Improved subjective sleep quality for night sleeps Improved perceived health status
Peacock et al., 1983 [91]	75	Sex not reported 32.8 years	Police officers	Rotating shift work	Change from 8 h 12 day shift cycle to a 12 h 8 day system	6 months	Blood pressure Subjective sleep duration (questionnaire) Subjective sleep quality (questionnaire)	Blood pressure decreased Increased subjective sleep duration Improved subjective sleep quality
Rosa et al., 1996 [92]	208	M = 190 F = 28 Young = 31.5 years Older = 50.0 years	Steel mill employees	Rotating shift work	Delaying shift start times by one hour	4 months	Objective sleep duration (actigraphy) Objective sleep quality (actigraphy) Subjective sleep duration (sleep diary) Subjective sleep quality (sleep diary)	Increased objective sleep duration on morning shift Increased subjective sleep duration on morning and night shift Deteriorated subjective sleep quality for evening and night shifts
Smith et al., 1998 [93]	72	M = 72 F = 0 39.1 years	Sewerage treatment plant employees	Rotating shift work	Change from slowly forward-rotating three shift (8 h) to continuous two shift (12 h)		Subjective sleep duration (sleep diary) Subjective sleep quality (sleep diary)	Improved subjective sleep quality for day sleep
Totterdell et al., 1992 [94]	71	Sex not reported Control = 29.5 years Intervention = 34.2 years	Police officers	Rotating shift work	Change from 8 h shifts with seven consecutive shifts to 8 h night shift and 10 h day	6 months	Subjective sleep duration (sleep diary) Subjective sleep quality (sleep diary)	Increased subjective sleep duration
Viitasalo et al., 2008 [95]	84	M = 84 F = 0 Rapidly forward-rotating = 47.0 years Flexible shift system = 37.0 years Control = 44.0 years	Maintenance employees	Rotating shift work	(I1) Change from backward-rotating to rapidly forward-rotating shift system (I2) Change from backward-rotating to more flexible system	7–8 months	Blood pressure BMI Blood lipids (TC, HDL-C, LDL-C) Blood glucose (fasting glucose) Self-report physical activity (International Physical Activity Questionnaire) Alcohol consumption (questionnaire) Nutritional intake (questionnaire) Subjective sleep quality (Basic Nordic Sleep Questionnaire)	(I1) Systolic blood pressure increased (12) Systolic blood pressure decreased
Watanabe et al., 2004 [96]	30	Sex not reported Single = 24.8 years Married = 32.5 years	Nurses	Rotating shift work	Change to a half day before night shift	4 day crossover	Objective sleep duration (actigraphy) Subjective sleep duration (sleep diary)	Increased objective sleep duration
Williamson et al., 1994 [97]	18	Sex not reported 23.8 years	Computer operators	Rotating shift work	Change from 8 h irregular roster to 12 h regular roster	12 months	Subjective sleep duration (sleep diary) Subjective sleep quality (sleep diary)	Increased subjective sleep duration after day shift and rest days Decreased subjective sleep duration following night shift
Williamson and Sanderson, 1986 [98]	16	Sex and age not reported	Controllers of emergency service	Rotating shift work	Change from weekly rotation 3 shift (7 days of same shift) to rapidly rotating roster of shifts with no more than three consecutive night shifts	5 months	Tobacco use (interview) Nutritional intake (food diary) Subjective sleep duration (sleep diary) Subjective sleep quality (sleep diary)	Improved subjective sleep quality
Yamada et al., 2001 [99]	205	M = 205 F = 0 Intervention group = 31.1 years Control group = 32.8	Clean room employees	Rotating shift work	Change from 8 h shift to 12 h shift	12 months	Blood pressure BMI	Significantly increased BMI

Note. BMI: body mass index; HDL-C: high-density lipoprotein cholesterol; LDL-C: low-density lipoprotein cholesterol; TC: total cholesterol.

**Table 4:** Studies investigating behavioural interventions.

Author Year	Sample Size	Sample Characteristics (Sex, Mean Age)	Occupation	Type of Shift Work at Baseline	Intervention Detail	Intervention Duration	Outcome (Measurement Used)	Results
Arora et al., 2007 [100]	58	Not reported	Medical interns	On-call rotating shift work	SAFER program 1 h lecture on Sleep, Alertness, and Fatigue Education in Residency	SAFER lecture = 60–90 min Intervention measurement = 1 month	Objective sleep (actigraphy)	No significant changes
Elliot et al., 2007 [101]	599	M = 579 F = 20 41.0 years	Firefighters	Rotating shifts	(I1) team-centred curriculum; leader ran 112 45 min team sessions using workbooks (I2) individual-centred motivational interviewing;	(I1) 11 × 45 min sessions (I2) 4 sessions Total study period = 12 months	BMI Physical activity (questionnaire) Nutritional Intake (questionnaire) Perceived health status (Modified RAND 36-Item Short-Form Health Survey)	(I1) Increased fruit and vegetable consumption Improved perceived health status (I2) Increased fruit and vegetable consumption Improved perceived health status
MacKinnon et al., [102] Follow up of Elliot et al., 2007	127	Not reported	Firefighters	Rotating shifts	Four-year follow up: (I1) team-centred curriculum; leader ran 112 45 min team sessions using workbooks (i2) individual-centred motivational interviewing	Four-year follow up	BMI Physical activity (questionnaire) Nutritional Intake (questionnaire) Perceived health status (Modified RAND 36-Item Short-Form Health Survey)	Improvements were not sustained at follow up
Harma et al., 1988 [103]	75	M = 0 F = 75 Training = 34.6 Control = 35.7	Nurses and nurses′ aids	Rotating shift work	Controlled physical activity training. Weekly training sessions (2–6 per week) were provided =	4 months	Subjective sleep quality (questionnaire) Subjective sleep duration (questionnaire)	Increased subjective sleep duration following evening shifts
Holbrook et al., 1994 [104]	38	M = 31 F = 7 M = 37.0 years F = 35.0 years	Police officers	Rotating shift work	Education of sleep hygiene practices	Education session = 1 h Total study period = 1 month	Subjective sleep quality (post-sleep inventory)	No significant changes
Kerin and Aguirre, 2005 [105]	Not reported	Not reported	Mine workers	Not reported	Workers and their spouses/partners attended the “Managing A Shiftwork Lifestyle” training workshop. Provided information on the solutions to the special challenges of shift work	6 weeks	Subjective sleep duration (sleep/wake logs)	Increased subjective sleep duration
Lee et al., 2013 [106]	21	M = 1 F = 20 45.5 years	Nurses	Night shift	Home-based cognitive-behavioural intervention—the Sleep Enhancement Training System for Shift Workers (SETS-SW)	8 week crossover	Objective sleep duration (actigraphy) Sleep efficiency (actigraphy) Subjective sleep quality (Pittsburgh Sleep Quality Index and General Sleep Disturbance)	Improved subjective sleep quality
Leedo et al., 2017 [107]	16	Sex not reported 40.9 years	Healthcare	Evening, night or weekend	Healthy meals and water provided at work	8 week crossover	BMI Self-report nutritional intake (dietary record)	Increased water intake
Lim et al., 2005 [108]	30	M = 30 F = 0 Control = 58.3 Intervention = 56.8	Not reported	Night shift	The experimental group performed an intermittent exercise at 10 min bouts (30 min per day), three days a week during 10 weeks	10 weeks	Blood Pressure BMI	No significant changes
Matsugaki et al., 2017 [109]	29	M = 0 F = 29 Supervised = 25.3 years Voluntary = 24.7 years	Nurses	Not reported	The supervised exercise group (SG; participants exercised under the supervision of a physical therapist (PT)) and the voluntary exercise group (VG; participants exercised without supervision). The study participants were asked to exercise twice/week for 12 weeks for 24 sessions	12 weeks	Blood pressure BMI Cholesterol (TC, LDL-C, HDL-C)	HDL-C increased
Morgan et al., 2011 [110]	110	M = 110 F = 0 44.4 years	Aluminium plant workers	Rotating shift work	The 3 month program involved one information session, program booklets, group-based financial incentives and an online component weight loss	14 weeks	Blood Pressure BMI Physical activity (Godin Leisure-Time Exercise Questionnaire) Dietary variables (questionnaire)	Blood pressure decreased Decreased BMI Increased physical activity Decreased sugary drink intake
Neil-Sztramko et al., 2017 [111]	20	M = 0 F = 20 42.2 years	17 = nurse/care aid 1 = service industry 1 = airline industry 1 = communications	19 = rotating, 1 = permanent night	Physical activity education 12 weekly sessions with physical activity coach (via phone)	12 weeks	BMI Physical Activity (actigraphy) Subjective sleep duration (Pittsburgh Sleep Quality Index) Subjective sleep quality (Pittsburgh Sleep Quality Index) Perceived health status (RAND 36-Item Short-Form Health Survey)	Decreased BMI Increased physical activity
Pylkkönen et al., 2018 [112]	49	Sex not reported Intervention = 37.9 years Control = 37.8 years	Truck drivers	Rotating shift work	The intervention group received a 3.5 h alertness management training followed by a two-month consultation period and motivational self-evaluation tasks two and 4–5 months after the training	4–5 months	Objective sleep duration (actigraphy) Subjective sleep duration (sleep log)	No significant changes
Smith-Coggins et al., 1997 [113]	6	M = 6 F = 0 34.0 years	Physicians in Emergency Department	Rotating shift work	Education session on sleep and sleep hygiene	1 month	Objective sleep duration (PSG) Subjective sleep duration (sleep diary) Subjective sleep quality (sleep diary)	No significant changes
Sullivan et al., 2017 [114]	1189	M = 1173 F = 16 43.6 years	US firefighters	Rotating shift work (including extended shifts)	Sleep hygiene education	1 year	Subjective sleep duration (questionnaire) Perceived health status (questionnaire)	No significant changes
Van Drongelen et al., 2014 [115]	502	M = 468 F = 34 40.9 years	Airline pilots	Not reported	The intervention group was given access to both the MORE Energy mobile application (app) with tailored advice and a website with background information. The control group was directed to a website with standard information about fatigue	3 months	Tobacco smoking (questionnaire) Physical activity (questionnaire) Alcohol consumption (questionnaire) Nutritional intake (questionnaire) Subjective sleep quality (Jenkins Sleep Scale) Subjective sleep duration (Pittsburgh Sleep Quality Index) Nutritional intake (questionnaire) Perceived health status (RAND 36-Item Short-Form Health Survey)	Increased physical activity Improved subjective sleep quality
Yeung et al., 2011 [116]	16	M = 7 F = 9 35.44 years	Manufacturing = 6 Customer service/management = 4 Nursing = 3 Public service = 1	Early morning shift	Participants advanced sleep time to 10 h before start of shift	2 week crossover	Objective sleep duration (actigraphy) Objective sleep efficiency (actigraphy) Subjective sleep duration (sleep diary) Subjective sleep quality (sleep diary)	Increased objective sleep duration Increased subjective sleep duration Improved subjective sleep quality

Note. BMI: body mass index; HDL-C: high-density lipoprotein cholesterol; LDL-C: low-density lipoprotein cholesterol; TC: total cholesterol; PSG: polysomnography.

**Table 5:** Studies investigating controlled light exposure interventions.

Author Year	Sample Size	Sample Characteristics (Sex, Mean Age)	Occupation	Type of Shift Work at Baseline	Intervention Detail	Intervention Duration	Outcome Measure (Measurement Used)	Results
Bjorvatn et al., 1999 [117]	7	M = 7 F = 0 38.9 years	Oil platform workers	Rotating shift work	Light box 10,000 lux for 30 min per day	4 nights offshore bright light 4 nights onshore bright light Total study period = 3 weeks	Subjective sleep duration (Karolinska sleep diary) Subjective sleep quality (Karolinska sleep/wake diary)	No significant changes
Bjorvatn et al., 2007 [118]	17	M = 16 F = 1 42.0 years	Oil platform workers	Rotating shift work	(i) Light box 10,000 lux for 30 min 4 night shifts and 4 day shifts (ii) Placebo capsule	Light period = 4 days on night shift 4 days of day shift Total study period = 6 weeks	Objective sleep duration (actigraphy) Objective sleep efficiency (actigraphy) Subjective sleep duration (sleep diary) Subjective sleep quality (sleep diary)	Reduction in objective sleep onset latency for night shift
Boivin et al., 2012 [119]	15	M = 6 F = 9 41.8 years	Nurses	Night shift	Intermittent bright light 3243 lux during first 6 h of each night shift Wore shaded goggles for 2 h following night shift	10 days	Objective sleep duration (nightcap or PSG) Objective sleep efficiency (nightcap or PSG)	Increased objective sleep duration
Budnick et al., 1995 [120]	13	M = 11 F = 2 Median age = 35.0 years	Industrial workers	Rotating shift work	Scheduled bright light (6000–12,000 lux)	3 months	Subjective sleep duration (sleep diary) Subjective sleep quality (sleep diary)	No significant changes
Costa et al., 1993 [121]	15	M = 0, F = 15 23.4 years	Nurses	Rotating shift work	Two consecutive night shifts exposed to short periods (4 × 20 min) of bright light (2350 Iux)	2 days control 2 days bright light	Subjective sleep duration (sleep diary)	Decreased subjective sleep duration between night shifts
Jensen et al., 2016 [122]	113	Sex not reported Intervention group = median age 43.0 years Control group = median age 42.0 years	Nurses	Rotating shift work	Intervention group worked in dynamic lighting designed to mimic natural daylight changes	10 days	Objective sleep duration (actigraphy) Sleep efficiency (actigraphy) Subjective sleep duration (sleep diary) Subjective sleep quality (sleep diary)	No significant changes
Lowden et al., 2004 [123]	18	M = 17 F = 1 36.2 years	Industrial truck production	Rotating shift work	20 min bright light (2500 lux) during breaks	4 week control 4 week bright light	Objective sleep duration (actigraphy) Sleep efficiency (actigraphy) Subjective sleep duration (sleep diary) Subjective sleep quality (sleep diary)	Increased objective sleep duration
Olson et al., 2020 [124]	33	M = 7 F = 26 32.7 years	Nurses	Rotating shift work	Light box (5500 lux) 40 min of light prior to night shift Sunglasses after night shift Sleep mask for sleeping after night shift	2 week control 2 week intervention	Subjective sleep duration (12 h sleep diary) Subjective sleep quality (Sleep Quality Scale) Objective physical activity (pedometer) Alcohol consumption (logbook)	Increased subjective sleep duration Improved subjective sleep quality
Sasseville et al., 2009 [125]	28	M = 13 F = 15 Summer = 42.4 years Winter = 37.2 years	Canada Post′s distribution centre	Fixed night workers	Blue-blockers glasses; (a) Summer group: just before leaving the workplace (b) Winter group: 2 h before the end of night shift	2 week control 2 week intervention	Objective sleep duration (actigraphy) Sleep efficiency (actigraphy)	Increased objective sleep duration Improved sleep efficiency
Sasseville et al., 2010 [126]	4	M = 4, F = 0 44.8 years	Sawmill workers	Rotating shift work	Blue-green light (200 lux) in workplace during shift Blue-blockers had to be worn outside from the end of the night shift until 16:00 h	1 week control 1 week light night shift 1 week light day shift	Objective sleep duration (actigraphy) Sleep efficiency (actigraphy)	Increased objective sleep duration
Simons et al., 2018 [127]	10	M = 3 F = 7 34.0 years	Intensive Care Unit nurses	Rotating shift workers (only on day shift for experiment)	Ceiling mounted dynamic light (1700 lux) in patients’ room	4 day control 4 day dynamic light	Subjective sleep quality (daily questionnaire) Subjective sleep duration (daily questionnaire) Perceived health status (World Health Organisation Quality of Life Short Form)	Deterioration in perceived health status
Tanaka et al., 2011 [128]	61	M = 0 F = 61 29.7 years	Nurses	Rotating shift work	Bright light (6666 lux) for 10 min prior to work in workplace	4 week control 4 week bright light	Alcohol consumption (self-report times per week) Subjective sleep quality (post-sleep visual analogue scale)	Improved subjective sleep quality
Thorne et al., 2010 [129]	10	M = 10 F = 0 Summer = 46.0 years Winter = 49.0 years	Offshore	Fixed night workers	Light box (3000 lux) and light-blocking glasses from waking until light treatment	21 days	Objective sleep duration (actigraphy) Objective sleep efficiency (actigraphy) Subjective sleep duration (sleep diary) Subjective sleep quality (sleep diary)	Increased objective sleep duration Improved sleep efficiency Decreased subjective sleep quality
Yoon et al., 2002 [130]	12	M = 0 F = 12 Age range = 21–24 years	Nurses	Night shift	(i) Room light (ii) Light box (4000–6000 lux) intermittent for four hours (iii) Light box with sunglasses worn next morning	4 days room light 4 days bright light 4 days light box with sunglasses	Objective sleep duration (actigraphy) Sleep efficiency (actigraphy) Subjective sleep duration (sleep diary) Subjective sleep quality (sleep diary)	(ii) Increased objective sleep duration Improved sleep efficiency (iii) Increased objective sleep duration Improved sleep efficiency

Note. PSG: polysomnography.

**Table 6:** Studies investigating complementary interventions.

Author Year	Sample Size	Sample Characteristics (Sex, Mean Age)	Occupation	Type of Shift Work at Baseline	Intervention Detail	Intervention Duration	Outcome Measure (Measurement Used)	Results
Chang et al., 2017 [131]	50	M = 0 F = 50 Intervention = 28.36 years Control = 30.55 years	Nurses	Rotating shift work	Intervention = 1 h lay down + 25 min aromatherapy massage once a week Control = once a week/4 times total 1 h lay down + music (no massage)	4 weeks	Sleep quality (Pittsburgh Sleep Quality Index) Objective sleep duration (Ezsleep—Electro-cardiogram signal collector)	Increased subjective sleep quality
Fazeli et al., 2020 [132]	12	M = 3 F = 9 Median age = 28.0	Medical residents	Rotating shift work	Intervention = 30 min massage Control = 30 min reading in chair	At least 18 days	Blood pressure	No significant change in intervention Blood pressure decreased in control group
McElligott et al., 2003 [133]	24	M = 3 F = 19 Control = 38.0 years Intervention = 34.0 years	Nurse	Rotating shift work	Touch therapy	4 weeks	Blood pressure	No significant result
Zadeh et al., 2018 [134]	36	M = 14, F = 22 Age 34.2 years	Nurse	Not reported	Transcutaneous Electrical Acupoint Stimulation (TEAS) (I1) Real TEAS points (I2) Sham points (I3) No intervention	4 weeks	Subjective sleep quality (Pittsburgh Sleep Quality Index)	(I1) Significantly improved subjective sleep quality (I2) Improved subjective sleep quality

## Data Availability

Data and reproducible R code available upon request.

## Supplementary Materials

The following are available online at https://www.mdpi.com/2624-5175/3/1/9/s1, S1: Search strategy. S2: Quality assessment and risk of bias with total score for each study by subgroup.
