# Supplementary material for: Non-Pharmacological Interventions to Improve Chronic Disease Risk Factors and Sleep in Shift Workers: A Systematic Review and Meta-Analysis
Source: Clocks Sleep. 2021 Jan 28;3(1):132–78. doi: 10.3390/clockssleep3010009 (PMC7930959; doi:10.3390/clockssleep3010009)
Supplement: Supplementary file 1 [file clockssleep-03-00009-s001.zip › Crowther et al_Supplementary Material 1.docx]

Supplementary Material 1

Search Strategy.

| **Database** | **Search strategy** |
| --- | --- |
| PsycINFO | AB (work AND shift OR shift work OR shiftwork OR shiftworker OR shift worker OR night shift) AND AB (intervention OR bright light OR light box OR goggle OR health promotion OR health intervention OR non-pharmacological intervention OR workplace education OR workplace program OR worksite intervention OR worksite program OR diet OR energy intake OR exercise OR physical activity OR occupational therapy OR shift change OR flexible working time OR positive psychology) AND AB (self-report health OR health outcomes OR risk factor OR chronic disease OR health OR circadian adaption OR BMI OR physical activity OR well being OR well-being) |
| MEDLINE | ab(work AND shift OR shift work OR shiftwork OR shiftworker OR shift worker OR night shift) AND ab(intervention OR bright light OR light box OR goggle OR health promotion OR health intervention OR non-pharmacological intervention OR workplace education OR workplace program OR worksite intervention OR worksite program OR diet OR energy intake OR exercise OR physical activity OR occupational therapy OR shift change OR flexible working time OR positive psychology) AND ab(self-report health OR health outcomes OR risk factor OR chronic disease OR health OR circadian adaption OR BMI OR physical activity OR well being OR well-being) |
| EMBASE | (**work**:ti,ab,kw AND **shift**:ti,ab,kw OR **'shift work'**:ti,ab,kw OR **shiftwork**:ti,ab,kw OR **shiftworker**:ti,ab,kw OR **'shift worker'**:ti,ab,kw OR **'night shift'**:ti,ab,kw) AND (**intervention**:ti,ab,kw OR **'bright light'**:ti,ab,kw OR **'light box'**:ti,ab,kw OR **goggle**:ti,ab,kw OR **'health promotion'**:ti,ab,kw OR **'health intervention'**:ti,ab,kw OR **'non-pharmacological intervention'**:ti,ab,kw OR **'workplace education'**:ti,ab,kw OR **'workplace program'**:ti,ab,kw OR **'worksite intervention'**:ti,ab,kw OR **'worksite program'**:ti,ab,kw OR **diet**:ti,ab,kw OR **'energy intake'**:ti,ab,kw OR **exercise**:ti,ab,kw OR **'physical activity'**:ti,ab,kw OR **'occupational therapy'**:ti,ab,kw OR **'shift change'**:ti,ab,kw OR **'flexible working time'**:ti,ab,kw OR **'positive psychology'**:ti,ab,kw) AND (**'self-report health'**:ti,ab,kw OR **'health outcomes'**:ti,ab,kw OR **health**:ti,ab,kw OR ‘risk factor**’**:ti,ab,kw OR ‘chronic disease’ **health**:ti,ab,kw OR **health**:ti,ab,kw OR **'circadian adaption'**:ti,ab,kw OR **bmi**:ti,ab,kw OR **'physical activity'**:ti,ab,kw OR **'well being'**:ti,ab,kw) |
| Cochrane Database | work AND shift OR shift work OR shiftwork OR shiftworker OR shift worker OR night shift in Title Abstract Keyword AND intervention OR bright light OR light box OR goggle OR health promotion OR health intervention OR non-pharmacological intervention OR workplace education OR workplace program OR worksite intervention OR worksite program OR diet OR energy intake OR exercise OR physical activity OR occupational therapy OR shift change OR flexible working time OR positive psychology in Title Abstract Keyword AND self-report health OR health outcomes OR health OR risk factor OR chronic disease OR circadian adaption OR BMI OR physical activity OR well being OR well-being in Title Abstract Keyword |
| PubMed | (((work[Title/Abstract] AND shift[Title/Abstract] OR shift work[Title/Abstract] OR shiftwork[Title/Abstract] OR shiftworker[Title/Abstract] OR shift worker[Title/Abstract] OR night shift[Title/Abstract])) AND (intervention[Title/Abstract] OR bright light[Title/Abstract] OR light box[Title/Abstract] OR goggle[Title/Abstract] OR health promotion[Title/Abstract] OR health intervention[Title/Abstract] OR non-pharmacological intervention[Title/Abstract] OR workplace education[Title/Abstract] OR workplace program[Title/Abstract] OR worksite intervention[Title/Abstract] OR worksite program[Title/Abstract] OR diet[Title/Abstract] OR energy intake[Title/Abstract] OR exercise[Title/Abstract] OR physical activity[Title/Abstract] OR occupational therapy[Title/Abstract] OR shift change[Title/Abstract] OR flexible working time[Title/Abstract] OR positive psychology[Title/Abstract])) AND (self-report health[Title/Abstract] OR health outcomes[Title/Abstract] OR health[Title/Abstract] OR risk factor[Title/Abstract] OR chronic disease[Title/Abstract] OR circadian adaption[Title/Abstract] OR BMI[Title/Abstract] OR physical activity[Title/Abstract] OR well being[Title/Abstract] OR well-being[Title/Abstract]) |
| Web of Science | **TOPIC:** (work AND shift OR shift work OR shiftwork OR shiftworker OR shift worker OR night shift) *AND* **TOPIC:** (intervention OR bright light OR light box OR goggle OR health promotion OR health intervention OR non-pharmacological intervention OR workplace education OR workplace program OR worksite intervention OR worksite program OR diet OR energy intake OR exercise OR physical activity OR occupational therapy OR shift change OR flexible working time OR positive psychology) *AND* **TOPIC:** (self-report health OR health outcomes OR health OR risk factor OR chronic disease OR circadian adaption OR BMI OR physical activity OR well being OR well-being) |
| CINAHL | AB ( work AND shift OR shift work OR shiftwork OR shiftworker OR shift worker OR night shift ) AND AB ( intervention OR bright light OR light box OR goggle OR health promotion OR health intervention OR non-pharmacological intervention OR workplace education OR workplace program OR worksite intervention OR worksite program OR diet OR energy intake OR exercise OR physical activity OR occupational therapy OR shift change OR flexible working time OR positive psychology ) AND AB ( self-report health OR health outcomes OR health OR risk factor OR chronic disease OR circadian adaption OR BMI OR physical activity OR well being OR well-being ) |
|  |  |
