# Supplementary material for: Non-Pharmacological Interventions to Improve Chronic Disease Risk Factors and Sleep in Shift Workers: A Systematic Review and Meta-Analysis
Source: Clocks Sleep. 2021 Jan 28;3(1):132–78. doi: 10.3390/clockssleep3010009 (PMC7930959; doi:10.3390/clockssleep3010009)
Supplement: Supplementary file 1 [file clockssleep-03-00009-s001.zip › Crowther et al_Supplementary Material 2.docx]

Supplementary Material 2

Quality assessment and risk of bias of included studies, by quality assessment subgroup and totals

| **First Author  (year of publication)** | **Reporting** | **External Validity** | **Internal validity, bias** | **Internal**  **validity, confounding** | **Power** | **Total score** |
| --- | --- | --- | --- | --- | --- | --- |
|  | (0-11) | (0-3) | (0-7) | (0-6) | (0-1) | (0-28) |
| Akerstedt (1978) | 5 | 2 | 5 | 2 | 0 | 14 |
| Amendola (2011) | 9 | 1 | 5 | 4 | 1 | 20 |
| Arora (2006) | 6 | 1 | 4 | 4 | 0 | 15 |
| Arora (2007) | 7 | 2 | 5 | 4 | 0 | 18 |
| Barton (1994) | 9 | 1 | 5 | 4 | 0 | 19 |
| Basner (2019) | 9 | 1 | 6 | 4 | 1 | 21 |
| Bjorvatn (1999) | 9 | 1 | 5 | 3 | 0 | 18 |
| Bjorvatn (2007) | 9 | 0 | 7 | 6 | 0 | 22 |
| Bøggild (2001) | 10 | 1 | 5 | 3 | 0 | 19 |
| Boivin (2012) | 10 | 0 | 4 | 1 | 0 | 15 |
| Bonnefond (2001) | 8 | 0 | 5 | 4 | 0 | 17 |
| Budnick (1995) | 7 | 1 | 4 | 3 | 0 | 15 |
| Chang (2017) | 10 | 0 | 6 | 5 | 0 | 21 |
| Costa (1993) | 9 | 0 | 5 | 4 | 0 | 18 |
| Czeisler (1982) | 4 | 0 | 4 | 3 | 0 | 11 |
| Di Milia (1998) | 9 | 0 | 5 | 4 | 1 | 19 |
| Elliot (2007) | 8 | 1 | 5 | 5 | 0 | 19 |
| Fazeli (2020) | 8 | 0 | 4 | 5 | 0 | 17 |
| Hakola (2002) | 7 | 0 | 5 | 3 | 0 | 15 |
| Hakola (2010) | 10 | 0 | 5 | 4 | 0 | 19 |
| Härmä (2006) | 10 | 0 | 4 | 3 | 0 | 17 |
| Härmä (1988) | 7 | 0 | 4 | 5 | 0 | 16 |
| Holbrook (1994) | 5 | 0 | 4 | 2 | 0 | 11 |
| Hornberger (1995) | 6 | 0 | 3 | 3 | 0 | 12 |
| Hornberger (1998) | 8 | 0 | 5 | 2 | 0 | 15 |
| Hossain (2004) | 8 | 0 | 5 | 4 | 0 | 17 |
| Jensen (2016) | 9 | 0 | 5 | 4 | 0 | 18 |
| Karhula (2020) | 8 | 0 | 3 | 0 | 0 | 11 |
| Karlson (2009) | 8 | 0 | 5 | 3 | 0 | 16 |
| Kerin (2005) | 1 | 0 | 1 | 1 | 0 | 3 |
| Knauth (1998) | 1 | 0 | 4 | 3 | 0 | 8 |
| Kobayashi (1997) | 7 | 2 | 4 | 3 | 0 | 16 |
| Lee (2014) | 10 | 0 | 5 | 4 | 0 | 19 |
| Leedo (2017) | 9 | 0 | 4 | 4 | 0 | 17 |
| Lim (2015) | 10 | 0 | 5 | 4 | 0 | 19 |
| Lowden (2004) | 9 | 0 | 5 | 4 | 0 | 18 |
| Lowden (1998) | 8 | 0 | 5 | 3 | 0 | 16 |
| MacKinnon (2010) | 8 | 1 | 5 | 3 | 0 | 17 |
| Matsugaki (2017) | 10 | 0 | 3 | 4 | 0 | 17 |
| McElligott (2003) | 4 | 0 | 3 | 3 | 0 | 10 |
| Mitchell (2000) | 9 | 3 | 4 | 3 | 0 | 19 |
| Morgan (2011) | 11 | 0 | 4 | 6 | 1 | 22 |
| Neil-Sztramko (2017) | 8 | 0 | 5 | 3 | 0 | 16 |
| Olson (2020) | 11 | 0 | 5 | 2 | 1 | 19 |
| Orth Gomer (1983) | 10 | 1 | 5 | 3 | 0 | 19 |
| Peacock (1983) | 6 | 0 | 5 | 2 | 0 | 13 |
| Pylkkönen (2018) | 10 | 0 | 4 | 5 | 1 | 20 |
| Rosa (1996) | 9 | 1 | 5 | 3 | 0 | 18 |
| Sasseville (2009) | 8 | 0 | 2 | 2 | 0 | 12 |
| Sasseville (2010) | 9 | 0 | 5 | 3 | 0 | 17 |
| Simons (2018) | 9 | 0 | 3 | 3 | 0 | 15 |
| Smith (1998) | 9 | 0 | 4 | 1 | 0 | 14 |
| Smith-Coggins (1997) | 8 | 0 | 5 | 5 | 0 | 18 |
| Sullivan (2017) | 9 | 1 | 5 | 4 | 1 | 20 |
| Tanaka (2011) | 11 | 1 | 4 | 6 | 0 | 22 |
| Thorne (2010) | 9 | 0 | 5 | 4 | 0 | 18 |
| Totterdell (1992) | 8 | 0 | 5 | 3 | 0 | 16 |
| van Drongelen (2014) | 10 | 1 | 4 | 5 | 0 | 20 |
| Vitasalo (2008) | 9 | 1 | 5 | 4 | 0 | 19 |
| Watanabe (2004) | 9 | 0 | 5 | 4 | 0 | 18 |
| Williamson (1994) | 8 | 0 | 3 | 1 | 0 | 12 |
| Williamson (1986) | 3 | 0 | 4 | 3 | 0 | 10 |
| Yamada (2001) | 10 | 0 | 4 | 2 | 0 | 16 |
| Yeung (2011) | 9 | 0 | 5 | 2 | 0 | 16 |
| Yoon (2002) | 6 | 0 | 4 | 1 | 0 | 11 |
| Zadeh (2018) | 9 | 0 | 5 | 3 | 0 | 17 |
|  |  |  |  |  |  |  |
| Mean | 8.12 | 0.36 | 4.47 | 3.33 | 0.11 | 16.39 |
| Standard Deviation | 2.11 | 0.65 | 0.93 | 1.27 | 0.31 | 3.58 |
